# Distinct chromatin signatures of DNA hypomethylation in aging and cancer

**DOI:** 10.1111/acel.12744

**Published:** 2018-03-05

**Authors:** Raúl F. Pérez, Juan Ramón Tejedor, Gustavo F. Bayón, Agustín F. Fernández, Mario F. Fraga

**Affiliations:** ^1^ Nanomedicine Group Nanomaterials and Nanotechnology Research Center (CINN‐CSIC) Universidad de Oviedo El Entrego, Asturias Spain; ^2^ Cancer Epigenetics Laboratory Institute of Oncology of Asturias (IUOPA) Hospital Universitario Central de Asturias (HUCA) Universidad de Oviedo Oviedo, Asturias Spain; ^3^ Cáncer Epigenetics Laboratory Fundación para la Investigación Biosanitaria de Asturias (FINBA) Instituto de Investigación Sanitaria del Principado de Asturias (ISPA) Oviedo, Asturias Spain

**Keywords:** aging, cancer, chromatin, DNA methylation, epigenetics, histone modification

## Abstract

Cancer is an aging‐associated disease, but the underlying molecular links between these processes are still largely unknown. Gene promoters that become hypermethylated in aging and cancer share a common chromatin signature in ES cells. In addition, there is also global DNA hypomethylation in both processes. However, the similarity of the regions where this loss of DNA methylation occurs is currently not well characterized, and it is unknown if such regions also share a common chromatin signature in aging and cancer. To address this issue, we analyzed TCGA DNA methylation data from a total of 2,311 samples, including control and cancer cases from patients with breast, kidney, thyroid, skin, brain, and lung tumors and healthy blood, and integrated the results with histone, chromatin state, and transcription factor binding site data from the NIH Roadmap Epigenomics and ENCODE projects. We identified 98,857 CpG sites differentially methylated in aging and 286,746 in cancer. Hyper‐ and hypomethylated changes in both processes each had a similar genomic distribution across tissues and displayed tissue‐independent alterations. The identified hypermethylated regions in aging and cancer shared a similar bivalent chromatin signature. In contrast, hypomethylated DNA sequences occurred in very different chromatin contexts. DNA hypomethylated sequences were enriched at genomic regions marked with the activating histone posttranslational modification H3K4me1 in aging, while in cancer, loss of DNA methylation was primarily associated with the repressive H3K9me3 mark. Our results suggest that the role of DNA methylation as a molecular link between aging and cancer is more complex than previously thought.

## 1 INTRODUCTION

Age is among the most important risk factors for cancer (de Magalhães, 2013; DePinho, 2000). However, the underlying molecular mechanisms governing this relationship are still poorly understood. Recent research has established polycomb‐target gene promoter hypermethylation as a common epigenetic characteristic of cancer (Schlesinger et al., 2007; Widschwendter et al., 2007). In this scenario, prior to alteration these promoters display an embryonic stem cell “bivalent chromatin pattern” consisting of the repressive histone mark H3K27me3 and the active mark H3K4me3 (Ohm et al., 2007). Genes affected by this process are associated with developmental regulation (Easwaran et al., 2012), implying a possible stem cell origin of cancer whereby aberrant hypermethylation could promote a continuously self‐renewing embryonic‐like state in cancer cells (Teschendorff et al., 2010). Interestingly, promoter hypermethylation of polycomb‐target genes was later described in aging blood (Rakyan et al., 2010; Teschendorff et al., 2010) and other tissue types such as mesenchymal stem cells (Fernández et al., 2015), ovary (Teschendorff et al., 2010), brain, kidney, and skeletal muscle (Day et al., 2013), findings which were also confirmed using whole‐genome bisulfite sequencing (Heyn et al., 2012).

In addition to aberrant locus‐specific DNA hypermethylation, tumoral cells are also globally hypomethylated as compared to their healthy counterparts. While this molecular alteration preferentially occurs at gene bodies, intergenic DNA regions, and repeated DNA elements (Ehrlich, 2009) and is proposed to be associated with chromosomal instability, reactivation of transposable elements, and loss of genomic imprinting, its precise functional role in cancer development is still poorly understood (Rodríguez‐Paredes & Esteller, 2011). Intriguingly, global loss of genomic DNA methylation has also been reported during the aging and senescence process (Cruickshanks et al., 2013; Fraga & Esteller, 2007). Whole‐genome bisulfite sequencing and methylation arrays have confirmed the global loss of DNA methylation in different human tissues including blood (Heyn et al., 2012), mesenchymal stem cells, and brain (Fernández et al., 2015). On the other hand, other important tissues such as skeletal muscle do not seem to become hypomethylated with aging (Zykovich et al., 2014).

Despite the interesting parallelism in aging and cancer recently reported with respect to hypermethylated DNA regions, the relationship between hypomethylated DNA sequences in these two processes has not been sufficiently studied. Moreover, recent analyses mainly performed in mouse tissue have failed to confirm global hypomethylation during the aging process (Cole et al., 2017; Hahn et al., 2017; Masser et al., 2017) and, to date, no study has provided a back‐to‐back and systematic comparison of the epigenetic changes that occur in aging and cancer. To address this issue, here we have analyzed DNA methylation changes and their associated chromatin patterns in a total of more than 2,300 healthy and tumoral samples obtained from differentially aged individuals, using HumanMethylation450 BeadChip data generated by The Cancer Genome Atlas (TCGA) consortium and other datasets (Bormann et al., 2016; Guintivano, Aryee & Kaminsky, 2013; Hannum et al., 2013). Our results confirmed the relationship between DNA hypermethylation in aging and cancer, but they also revealed important differences in DNA hypomethylation changes in the two processes that might be important to understand the possible role of DNA methylation as a molecular link between decline related to aging and tumor development.

## 2 RESULTS

### 2.1 DNA methylation profiling in aging and cancer

**Figure 1:**
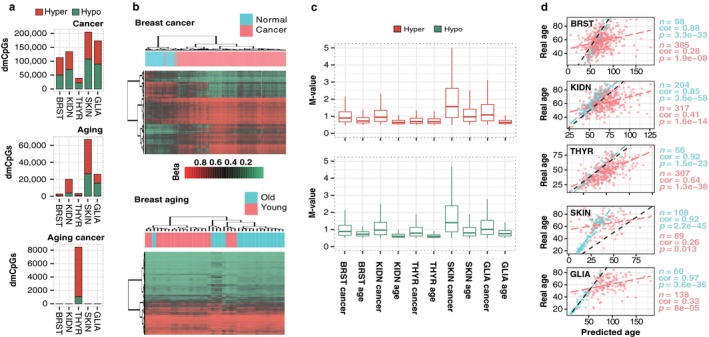
DNA methylation changes in aging and cancer. (a) Stacked barplots indicating total number of dmCpGs detected in cancer, aging, and aging cancer tissues. (b) Hierarchical clustering and heatmaps including the 1,000 most significant dmCpGs for breast cancer and aging analyses. Beta‐values of DNA methylation are displayed from zero (green) to one (red). (c) Boxplots comparing the magnitude of M‐values of methylation changes in cancer and aging. All differences are statistically significant (Wilcoxon tests, all *p* < .05, Table S3). (d) Scatterplots indicating a correlation of chronological age with Horvath's predicted age in normal and cancer samples. Pearson's product‐moment correlation coefficient (cor) is indicated, and linear fit lines are added to help with data interpretation

To identify DNA methylation changes in aging and cancer, we collected DNA methylation data obtained with the HumanMethylation450 BeadChip (Illumina) (see Section 4) and compared the DNA methylation status of a total of 361,698 CpG sites across 1,762 samples corresponding to healthy and tumoral tissues obtained from differentially aged patients with breast, kidney, thyroid, skin, and brain tumors (see Tables 1 and S1 for extended information). Using an empirical Bayes moderated *t* test (see Section 4), we identified a high number of autosomal differentially methylated CpGs (dmCpGs; FDR < 0.05) between normal and tumoral samples, while a lower and more variable number of aging‐related dmCpGs between young and old samples was found (Table 1; Figure 1a and Table S2 for additional information). Hierarchical clustering of samples using the dmCpGs enabled us to distinguish between tumoral and control samples with more efficiency than young and old samples (Figure 1b and Figure S1). On the whole, whereas cancer‐related DNA methylation changes had no dominance of either hyper‐ or hypomethylation, aging‐related changes tended toward DNA hypermethylation, and showed a much more variable and tissue type‐dependent magnitude of change. Globally, methylation changes were found to be more pronounced in cancer than in aging (Figure 1c and Table S3, Wilcoxon tests; all *p *<* *.05), while comparison of hyper‐ vs. hypomethylation changes was variable and disease and tissue type dependent. Intriguingly, most tumors obtained from differentially aged patients did not show significant age‐associated DNA methylation changes, with the exception of thyroid cancer (Table 1, Figure 1a, bottom panel and Table S2). Furthermore, analyses employing Horvath's predictor revealed that thyroid cancer had the highest correlation of real‐vs.‐predicted age across all the cancer types in our dataset (Figure 1d).

**Table 1:** Description of sample groups and dmCpGs obtained in the analyses

		BRST				KIDN				THYR				SKIN				GLIA			
	Total samples	483				521				363				197				198			
		Normal		Tumor		Normal		Tumor		Normal		Tumor		Normal		Tumor		Normal		Tumor	
	Group	98		385		204		317		56		307		108		89		60		138	
	Source	TCGA		TCGA		TCGA		TCGA		TCGA		TCGA		E‐MTAB 4385		TCGA		GSE 41826		TCGA	
Cancer	Total Samples	483				371				363				197				198			
		Normal		Tumor		Normal		Tumor		Normal		Tumor		Normal		Tumor		Normal		Tumor	
	Samples group	98		385		154		217		56		307		108		89		60		138	
	Age mean	57.6		57.7		62.5		61.3		45.8		47		47.2		64.4		32.6		59.9	
	Age range	28–90		26–90		31–90		26–90		15–81		15–89		18–78		24–90		13–79		21–85	
	Gender (M,F)	0	98	4	381	106	48	141	76	14	42	74	233	0	108	54	35	29	31	79	59
	DMPs Total	113,314				134,672				38,593				205,134				173,871			
	DMPs hyper	63,196				65,195				16,561				97,362				84,334			
	DMPs hypo	50,118				69,477				22,032				107,772				89,537			
Aging	Total Samples	98				204				56				108				60			
		Young		Old		Young		Old		Young		Old		Young		Old		Young		Old	
	Samples group	20		18		23		22		19		18		16		15		20		19	
	Age mean	37.7		81.1		43.3		82.0		28.3		66.7		19.9		73.5		16.4		51.8	
	Age range	28–44		75–90		31–49		78–90		15–34		55–81		18–22		71–78		13–21		43–79	
	Gender (M,F)	0	20	0	18	15	8	13	9	4	15	7	11	0	16	0	15	12	8	13	6
	DMPs Total	2,588				20,019				3,216				66,977				25,849			
	DMPs hyper	2,077				16,227				2,079				40,640				10,533			
	DMPs hypo	511				3,792				1,137				26,337				15,316			
Aging cancer	Total Samples	385				317				307				197				198			
		Young		Old		Young		Old		Young		Old		Young		Old		Young		Old	
	Samples group	28		26		29		28		28		27		30		29		28		25	
	Age mean	35.1		83.3		40.2		82.1		22.6		75.2		48.4		79.4		41.4		76.3	
	Age range	26–40		79–90		26–46		78–90		15–27		70–89		24–58		73–90		21–51		72–85	
	Gender (M,F)	0	28	1	25	19	10	18	10	7	21	10	17	20	10	14	15	13	15	12	13
	DMPs Total	–				–				8,480				–				–			
	DMPs hyper	–				–				7,429				–				–			
	DMPs hypo	–				–				1,051				–				–			

### 2.2 Genomic distribution of dmCpGs in aging and cancer

**Figure 2:**
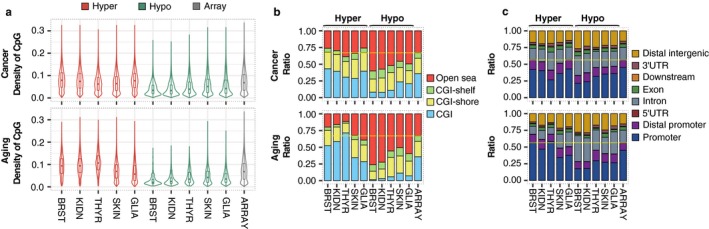
Similarities and disparities in the genomic distribution of methylation changes in aging and cancer. (a) Violin plots showing the distribution of CpG density for cancer and aging hyper‐ and hypomethylated dmCpGs and the background sites in the Infinium HumanMethylation450 microarray. (b) Stacked barplots indicating relative distribution of differentially methylated CpGs according to their CpG island status. A dashed yellow line separates CpG island associated locations from open sea. (c) Stacked barplots indicating relative distribution of differentially methylated CpGs according to their gene location status. A dashed yellow line separates promoter associated locations from the rest

The study of the genomic distribution of the dmCpGs revealed that hypomethylated CpG sites followed a similar disease and tissue‐independent trend, being preferentially found at low‐density CpG DNA regions interrogated by the array in both cancer and aging (average median difference compared to array 49%, Wilcoxon tests; all *p *<* *.001) (Figure 2a; see also Table S4). Consequently, with respect to the array, these hypomethylated CpG sites were enriched at open sea locations and intronic and intergenic regions (Fisher's tests; all *p *<* *.001, all odds ratios (ORs) >2.25, >1.34, and >1.21, respectively, except nonsignificant thyroid aging) while impoverished at CpG islands and gene promoters (Fisher's tests; all *p *<* *.001, all ORs<0.43 and <0.64, respectively) (Figure 2b,c; Figure S2 and Table S5). Density of hypermethylated CpG sites in cancer was variable but comparable to background array density (average median difference <±12%, Wilcoxon tests; all *p *<* *.001), whereas a noticeably high CpG density was found for breast, kidney, and thyroid in the aging context (average median difference 44%, Wilcoxon tests; all *p *<* *.001). Consequently, these dmCpGs were enriched at CpG islands and gene promoters (Fisher's tests; all *p *<* *.001, all ORs>1.89 and >1.08, respectively).

Comparative enrichment analysis confirmed that DNA hypomethylation in aging and cancer mainly occurred at low CpG density DNA regions located at introns, open sea, and intergenic DNA regions, while hypermethylation distribution was more irregular and more similar to array distribution. Nonetheless, a common and strong tendency was found when comparing hyper‐ to hypomethylation changes in both aging and cancer, whereby hypermethylation changes always occurred in regions with a higher CpG density than did hypomethylation changes (average median difference 51%, Wilcoxon tests; all *p *<* *.001) (Figure 2a and Table S4), resulting in strong differences in local enrichments at CpG islands and open sea locations, as well as gene promoters and intergenic regions, in most cases (Figure 2b,c; Figure S3 and Table S5), with this effect being even more pronounced for the aging dmCpGs.

### 2.3 Tissue type‐independent DNA methylation changes in aging and cancer

**Figure 3:**
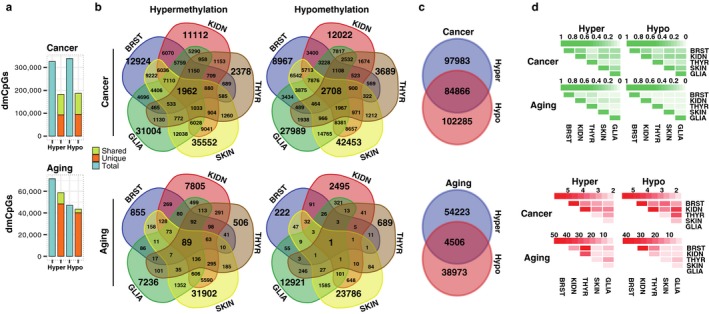
DNA methylation signatures of aging and cancer. (a) Stacked barplots indicating, in blue, the total number of hyper‐ and hypomethylated dmCpGs detected between all of the tissues analyzed in cancer and aging. Of these, the proportion of dmCpGs not shared between any tissues (unique, orange) or those shared by two or more tissues (shared, green) is shown in the adjacent barplot. (b) Venn diagrams depicting the number of differentially hyper‐ and hypomethylated CpGs in aging and cancer shared by the different tissues. (c) Venn diagrams showing the number and overlap of total nonredundant hyper‐ and hypomethylated dmCpGs detected in cancer and aging. (d) Heatmaps showing pairwise comparisons between sets of probes: in green, Jaccard Indices; in red, odd ratios (all enrichment Fisher's tests *p* < .001)

To determine the effect of tissue type on the DNA methylation changes during aging and cancer, we compared the previously identified dmCpG sites for each of the tissues. In cancer, ~50% of hyper‐ and hypomethylated CpGs were common to at least two different tumor types (Figure 3a), with 1,962 (1.1%) hyper‐ and 2,708 (1.5%) hypomethylated CpG sites being common to all five tumor types analyzed (Figure 3b and Table S6). In contrast, the overlap between dmCpGs in aging across different tissues was considerably reduced. Indeed, only 18% of the hyper‐ and 8% of the hypomethylated CpG sites were common to at least two tissue types and only 89 (0.15%) hyper‐ and 1 (0.002%) hypomethylated CpG sites were common to all five tissue types analyzed (Figure 3a,b and Table S6). However, statistical analyses of the pairwise overlaps between the different sets of probes showed overall enrichment in every case, especially for aging (Figure 3d, Fisher's tests; all *p *<* *.001, Table S7). This over‐enrichment was also revealed through a simulation of a random sampling of probes from the array (Figure S4a). Taken together, these results suggest that both cancer and aging manifest tissue‐independent changes in DNA methylation.

We also identified a subset of dmCpG sites in aging and cancer that could potentially be either hyper‐ or hypomethylated depending on the tissue type involved (Figure 3c) and showed substantial under‐enrichment (Fisher's test *p *<* *.001 for both, ORs = 0.65 and 0.56; expected hypergeometric means, EHMs = 94,610 and 7,060; and Jaccard indices, JIs = 0.30 and 0.05, respectively). Interestingly, when examining dmCpGs shared by two or more tissues (Figure S4b), this under‐enrichment became more pronounced such that CpGs that were thus affected in more than one tissue were less likely to behave differently in other tissues.

### 2.4 Similar chromatin signatures of DNA hypermethylation in aging and cancer

**Figure 4:**
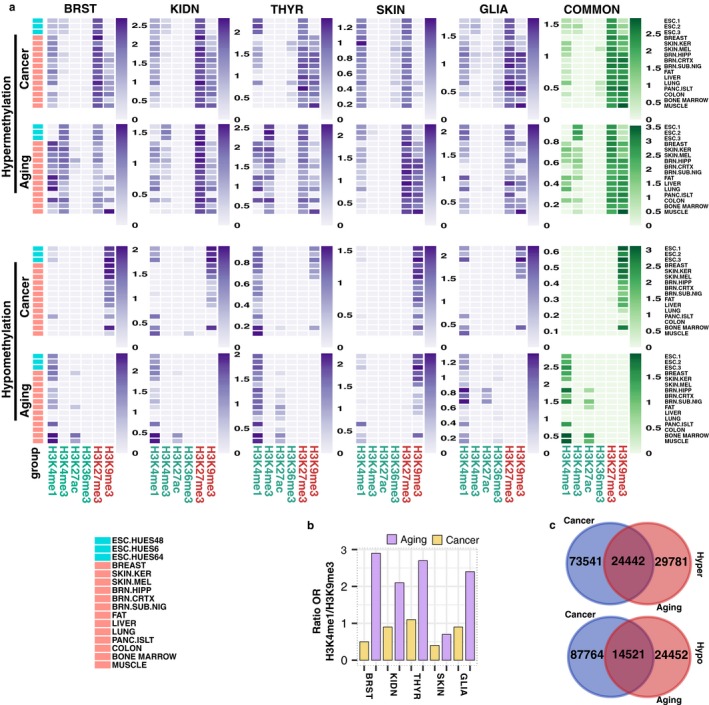
Distinct chromatin signatures of hyper‐ and hypomethylation in aging and cancer. (a) Heatmaps depicting significant (*p* < .05) over‐enrichment of hyper‐ and hypomethylated dmCpG sites with different histone marks in aging and cancer, in a selection of 16 cell and tissue types (see Table S8 for 98 full cell and tissue types). Color code indicates the significant enrichment based on log2 odds ratio (OR). Common signatures are calculated from hyper‐ and hypomethylated dmCpGs shared between five tissues for cancer (1,962 and 2,708 probes, respectively) or three tissues for aging (904 and 106 probes, respectively) (see Table S6 for CpG lists). (b) Barplots indicating the ratio of OR for the H3K4me1/H3K9me3 marks associated with hypomethylated dmCpGs in aging and cancer. The ratio is calculated by taking the mean of OR of those tracks with significant over‐enrichment for each histone mark and dividing the obtained numbers. (c) Venn diagrams showing the number and overlap of total nonredundant hyper‐ and hypomethylated dmCpGs detected in cancer and aging. dmCpGs that were only hypermethylated or only hypomethylated between all tissues were chosen for the comparison

To identify possible chromatin marks associated with hypermethylated CpG sites in aging and cancer, we compared the hypermethylated CpG sites identified in this study with previously published ENCODE and NIH Roadmap Epigenomics ChIP‐seq data on the histone modifications H3K4me1, H3K4me3, H3K27ac, H3K36me3, H3K27me3 and H3K9me3 across 98 different cell and tissue types (see Section 4). The results confirmed an enrichment of hypermethylated CpG sites in repressive histone modifications H3K27me3 and H3K9me3 and active histone modifications H3K4me1 and H3K4me3 in both in aging and cancer (Figure 4a, upper panel; Table S8), with the H3K27me3 mark being the most consistent enrichment across all of the analyses. Notably, these similarities became more pronounced when examining dmCpGs shared by all five tissue types in cancer, or three of the tissue types in aging (low numbers of common probes, due to tissue‐specificity of aging dmCpGs, hindered analysis of dmCpGs shared by more tissues). Interestingly, the embryonic stem cell signature was comparable to other tissue signatures, although, when present, the H3K4me3 mark was more evident in the aging context. Collectively, these results suggest that chromatin signatures of DNA hypermethylation are similar in aging and cancer.

### 2.5 Distinct chromatin signatures of DNA hypomethylation in aging and cancer

To determine whether the chromatin signatures of DNA hypomethylation were also similar in aging and cancer, we compared the hypomethylated CpG sites identified in our study with data from the same histone modifications as described in the earlier analyses. Interestingly, the results showed that hypomethylated CpG sites in cancer were enriched in the repressive H3K9me3 histone modification, while in aging, hypomethylated CpGs were more enriched in the activating histone mark H3K4me1 (Figure 4a, lower panel; Table S8). There were though exceptions to this general trend: hypomethylated CpG sites in thyroid tumors were also enriched at H3K4me1, and hypomethylated DNA sequences in aged skin were mainly co‐associated with H3K9me3‐marked DNA regions. Nevertheless, the ratio H3K4me1/H3K9me3 was always higher in aging than in cancer (Figure 4b). Moreover, when analyzing the dmCpGs shared by all five tissues in cancer or at least three tissues in aging, these distinct chromatin signatures became much more evident. In sum, these results indicate that, in contrast to DNA hypermethylation, chromatin signatures of DNA hypomethylation in aging and cancer differ considerably.

After deriving the chromatin signatures, we performed validation analyses on two additional datasets: the first related to tissue from TCGA control and lung adenocarcinoma and the second to whole blood from the classical Hannum et al. (2013) dataset (Figure S5; see Table S9 for additional information). Interestingly, we were unable to find aging‐related methylation changes in normal lung tissue using our pipeline. The magnitude and distribution of the hyper‐ and hypomethylation changes in lung cancer and whole blood aging followed the same trend as observed for the other datasets (Figure S5a, see Table S2 for a list of dmCpGs). The histone enrichment analyses revealed the same hypermethylation signature previously found for cancer and aging, and very clear and different hypomethylation signatures of H3K9me3 for lung cancer and H3K4me1/3 for whole blood aging (Figure S5b and Table S8).

Finally, we compared the overlap between either hypermethylated or hypomethylated CpGs across tumors and their corresponding age‐related tissues (Figure 4c). This approach revealed that the overlap between hypermethylated CpGs (24,442 CpGs) was higher than expected by chance (Fisher's test *p *<* *.001, OR = 2.61; EHM = 14,689; JI = 0.20) (Figure 4c, upper panel). However, despite the overlap between hypomethylated CpGs (14,521) also being slightly higher than expected (Fisher's test *p *<* *.001, OR = 1.60; EHM = 11,021; JI = 0.12) (Figure 4c, lower panel), the overall trend observed in this case was weaker than for the hypermethylated CpGs. Furthermore, most of the hypomethylated probes shared by cancer and aging belonged to skin dmCpGs, providing evidence for its similar cancer and aging hypomethylation signatures. Removing skin tissue from the analysis (Figure S6) caused the observed over‐enrichment to disappear in the case of DNA hypomethylated probes, although it remained in the hypermethylation scenario (Fisher's tests, both *p *<* *.001, ORs = 0.8 and 3.0; EHMs = 5,357 and 7,568; JIs = 0.04 and 0.12, respectively), reinforcing the observed overlapping differences between hyper‐ and hypomethylated probes.

### 2.6 Functional characterization of differentially methylated sites in aging and cancer

To determine the possible functional consequences and genomic coincidence of the different histone marks of DNA hyper‐ and hypomethylation in aging and cancer, we performed an enrichment analysis of NIH Roadmap and ENCODE Hidden Markov Model (HMM) defined “chromatin states” across the same 98 human cell and tissue types used in the previous analyses (see Section 4). In total, 18 states were used for the segmentation of the genome, which were then grouped to highlight predicted functional elements.

**Figure 5:**
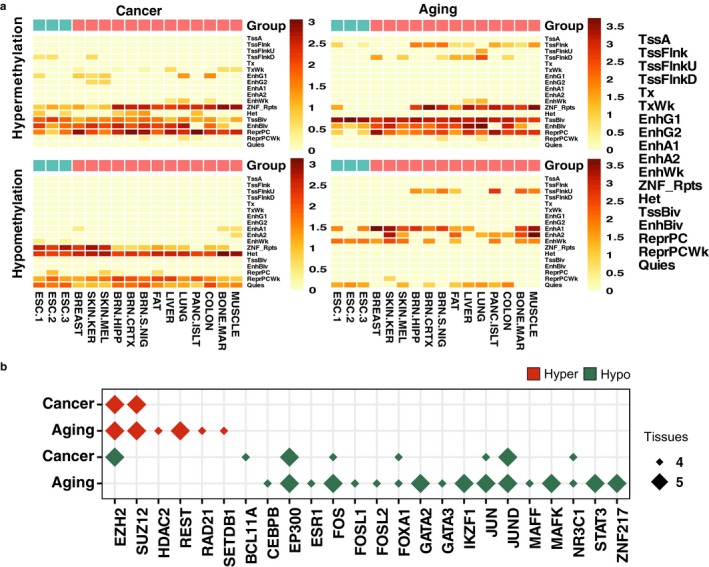
Differential chromatin state signatures of hyper‐ and hypomethylation in aging and cancer. (a) Heatmaps displaying significant (*p* < .05) over‐enrichment of hyper‐ and hypomethylated dmCpG sites with different chromatin states in aging and cancer, in a selection of 16 cell and tissue types (see Figure S7 for tissue‐specific signatures, and Table S10 for 98 full cell and tissue types). Color code indicates the significant enrichment based on log2 odds ratio (OR). Common signatures are calculated from hyper‐ and hypomethylated dmCpGs shared between five tissues for cancer (1,962 and 2,708 probes, respectively) or three tissues for aging (904 and 106 probes, respectively) (see Table S6 for CpG lists). (b) Panel indicating significant (*p* < .05) transcription factor enrichment at hyper‐ and hypomethylated dmCpG sites in aging and cancer that occurred in at least four or five tissues (see Figure S8 for tissue‐specific results and Table S11 for full data). Only the most representative transcription factors (those that appeared as significantly over‐enriched in at least three tracks or with an OR > 3 in any track) were selected for data representation

As suggested by the earlier chromatin signature analyses, hypermethylated CpGs in both aging and cancer were enriched in states associated with bivalent chromatin domains (i.e., those formed by the combination of repressive histone mark H3K27me3 and activating histone marks H3K4me1/3), polycomb repressive domains, and repeat/ZNF genes. These patterns became more evident when examining the dmCpGs shared by all five cancer tissues or at least three aging tissues (Figure 5a; see Figure S7 for tissue‐specific signatures and Table S10 for full data in all 98 cell and tissue types). Hypomethylated CpG sites in cancer were enriched in chromatin states associated with heterochromatin and repeat/ZNF gene domains and, to a lesser extent, polycomb repressive domains. In contrast, DNA hypomethylation in aging was primarily associated with chromatin states related to DNA enhancers. Again, these marks were more pronounced in aging dmCpGs shared by at least three tissues. As occurred with chromatin signatures, hypomethylation chromatin state differences were weaker in skin and thyroid, albeit that the ratio of change of chromatin states always followed the same behavior (data not shown). Collectively, these results support the notion that DNA hypomethylation might have a different functional role in aging as compared to cancer.

To increase our understanding of the functional context of the chromatin signatures characterized, we compared the dmCpG sites identified in this study with publicly available ENCODE ChIP‐seq data on transcription factor binding sites in 689 datasets corresponding to 188 transcription factors across 91 different cell types (see Section 4) (Figure 5b; see Figure S8 and Table S11 for full tissue‐specific results). As expected, hypermethylated CpGs in aging and cancer were associated in all tissues with the presence of EZH2 and SUZ12, components of the polycomb complex which directly deposits the H3K27me3 mark. Interestingly, aging hypermethylation dmCpGs were specifically associated with other types of transcription factors in various tissues, such as REST, HDAC2, RAD21, and SETDB1. Transcription factor enrichment at hypomethylated dmCpG sites was more heterogeneous than, but different from that of hypermethylated sites. Enrichment of similar factors was found for cancer and aging, for example, EP300, FOS, and JUN, among others. As observed before, specific aging enrichment was found, such as GATA2/3. In this case, aging hypomethylated dmCpG sites tended to display a more marked enrichment of most of the cancer hypomethylation factors and, additionally, revealed the presence of other family‐ or function‐related proteins, like FOSL1/2, MAFF, MAFK, and STAT3. When examining enrichment at common dmCpG sites shared by different tissues in cancer and aging, the initial observations were further confirmed (Figure S9).

**Figure 6:**
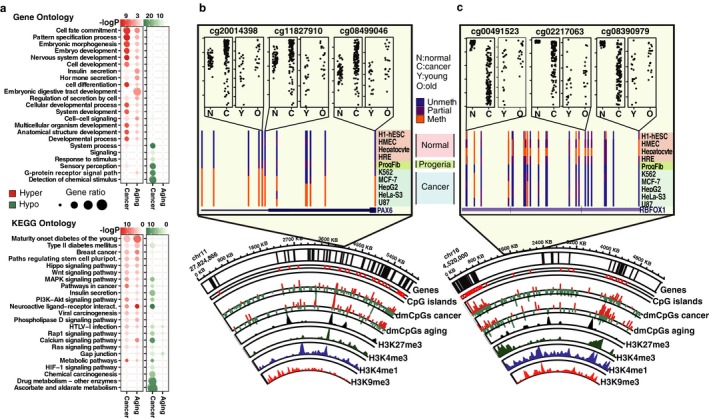
Dissimilar functional context of differentially methylated CpGs in aging and cancer. (a) Panels indicating gene and KEGG pathway ontology enrichment for common hyper‐ and hypomethylated dmCpGs shared between five tissues for cancer (1,962 and 2,708 probes, respectively) or three tissues for aging (904 and 106 probes, respectively) (see Table S6 for CpG lists and Figure S9). Color code indicates the significance of the over‐enrichment based on log10 *p*‐value. Size of circles indicates gene ratio, calculated as the ratio of a number of identified hits with respect to the total number of components in a given ontology. (b) and (c) Circular representation of illustrative genomic locations indicating hyper‐ (red) and hypomethylated (green) dmCpG sites in cancer and aging. Inner tracks display chromatin marks (H3K27me3, H3K4me3, H3K4me1, and H3K9me3, respectively), corresponding to ChIP‐seq peak data from ESC E014 NIH Roadmap track (circos, lower panel). Two examples of hypo‐ and hypermethylated genes are highlighted from the circular figures, displaying methylation data obtained from ENCODE/HAIB methyl450 track set from UCSC Genome Browser (hg19) and plots of methylation values extracted from a representative example of our aging and cancer analyses corresponding to glia tissue (upper panel, depicted CpGs also displayed a similar trend in kidney and skin analyses, data not shown)

Gene ontology analyses (Figure 6a; Table S12; see Figure S10 for tissue‐specific results) revealed that hypermethylated CpGs in both processes belonged to genes that were mainly related to developmental functions. While genes containing hypomethylated CpGs in cancer were associated with extracellular signaling, those for aging were, in general, much less enriched in any gene ontology. In the case of KEGG pathways (Figure 6a; Table S12 and Figure S10), hypermethylated CpGs in both cancer and aging shared enrichment for several ontologies, many related to cell metabolic and signaling pathways. In this respect, hypomethylated CpGs in cancer had some ontologies in common, while others were specific. Once again, aging hypomethylated CpGs exhibited much less enrichment in any function.

To exemplify the similarities and disparities observed for DNA methylation in aging and cancer, we focused on a number of significant dmCpGs from two particular genomic regions, located in chromosomes 11 and 16 (Figure 6b,c). We observed a substantial correlation between bivalent posttranslational histone modifications, especially H3K27me3 and H3K4me1/3, and the presence of hypermethylated probes in aging and cancer. On the other hand, DNA hypomethylated regions were more frequently located near H3K9me3 or H3K4me1 peaks (bottom panel Figure 6b,c) as outlined in our previous histone enrichment analyses. A detailed inspection of the common genes with most abundant dmCpGs in aging and cancer revealed a similar trend toward DNA hypermethylation at the boundaries of the gene *PAX6* (Figure 6b, top panel). Interestingly, a representative set of cancer cell lines, as well as fibroblasts derived from patients with Hutchinson‐Gilford progeria, also display higher levels of DNA methylation when compared to normal cells in these differentially methylated regions (Figure 6b, middle panel). On the contrary, the abovementioned pattern was mainly reversed in the case of the *RBFOX1* gene, located in a region which was preferentially hypomethylated in cancer (Figure 6c, top and middle panel).

### 2.7 Correlations between CpG methylation and gene expression in aging and cancer

**Figure 7:**
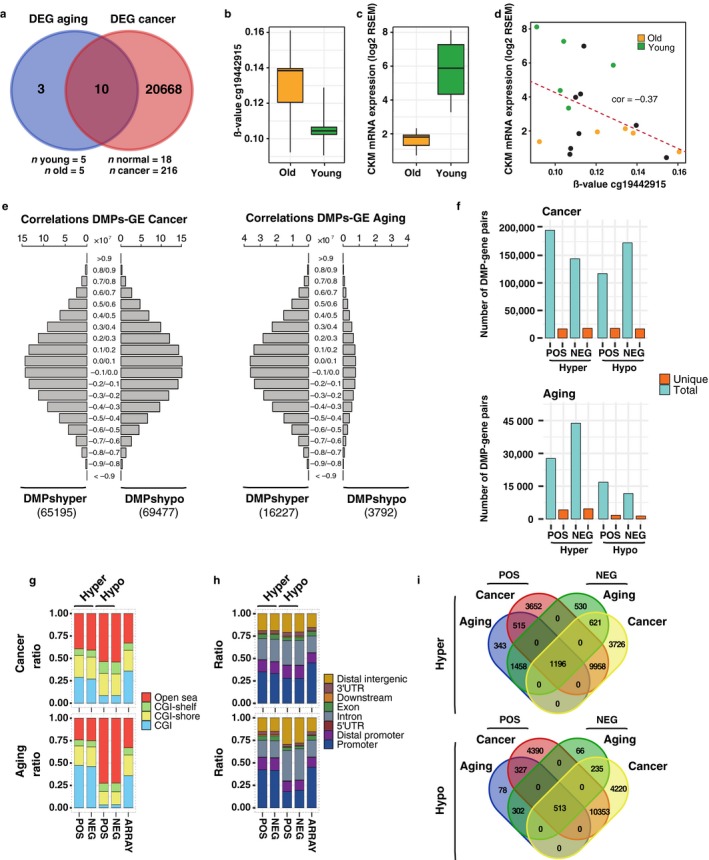
Relationships between DNA methylation and gene expression in aging and cancer. (a) Venn diagrams illustrating the overlap between DEGs in aging and cancer in the KIRC dataset (see Table S13 for DEG lists). (b) Boxplot depicting the DNA methylation β‐values of the CpG cg19442915 in old and young individuals (*n* = 5) from the KIRC aging condition. (c) Boxplot showing the gene expression values (RSEM) of the CKM gene in old and young individuals (*n* = 5) from the aging condition of the KIRC dataset. (d) Scatterplot showing the Spearman correlation between DNA methylation (cg19442915) and gene expression (CKM gene) in 18 normal kidney samples. Colored dots indicate old or young individuals used for the aforementioned aging comparisons. (e) Histograms representing the number of pairwise correlations that are contained in a given correlation window (from 1 to −1) obtained as the result of computing the correlation between β‐values of dmCpGs identified in cancer or aging and gene expression levels (RSEM) of genes expressed in the KIRC dataset. The number of dmCpGs used for each of the comparisons is indicated at the bottom. (f) Barplots depicting the number of total (blue) and unique (orange) dmCpG‐gene pairs identified in the previous analysis which displayed correlation scores above 0.9 (pos) or below −0.9 (neg) in cancer (top) or in aging (bottom) conditions. (g) Stacked barplots indicating relative distribution of unique dmCpGs obtained from the previous correlations according to their CpG island status. (h) Stacked barplots indicating relative distribution of unique dmCpGs obtained from the previous correlations according to their gene location status. (i) Venn diagrams illustrating the overlap between dmCpGs identified in aging and in cancer which displayed strong positive or negative correlations (>0.9 or <−0.9) with genes expressed in the normal kidney dataset

Lastly, we looked at the possible impact in the control of gene expression of the methylation changes previously found. To address this issue, we focused on kidney tissue (KIRC) as this TCGA dataset displayed a reasonable number of control and cancer patients with paired methylation and gene expression data. We initially performed differential gene expression analyses comparing young vs. old or normal vs. tumoral kidney samples (Figure 7a and Table S13). These results allowed us to identify a total of 13 and 20,678 differentially expressed genes (DEGs) in aging and cancer conditions, respectively. The majority of the aging DEGs were also found in cancer, including, for example, the *CKM* gene, which contained a dmCpG in the proximity of its promoter (Figure 7b), was differentially expressed in both processes (Figure 7c) and displayed a considerable negative correlation between DNA methylation and gene expression in normal kidney (Spearman *r* = −.37, Figure 7d). To further explore the potential relationships between CpG methylation and gene expression in these processes, and due to the reduced number of DEGs observed in the aging context, we decided to perform all potential pairwise correlations between DNA methylation and gene expression using cancer‐ or aging‐related dmCpGs and genes expressed in a subset of normal kidney tissue samples (*n* = 18). This approach enabled us to quantify the extent to which CpGs whose methylation status changes in cancer and aging originally influence gene expression in normal tissue.

We computed a total of 2.58e^09^ and 3.84e^08^ correlations between cancer‐ and aging‐related dmCpGs, respectively, and genes expressed in the normal KIRC dataset (Figure 7e). Despite the considerable difference in a number of dmCpGs between cancer and aging, when compared to the total possible number of correlations, we found similar percentages of strong correlations between DNA methylation and gene expression in both processes (Table S14). Moreover, these proportions were also higher than those observed when sampling random probes from the array and computing their correlations (see Figure S11). These results indicate that both cancer‐ and aging‐related dmCpGs are enriched in CpGs that can influence, to some extent, gene expression in kidney tissue.

A more detailed inspection of the strongest correlations (>= 0.9 or <= −0.9) identified in these datasets revealed that, in cancer, the number of unique dmCpG‐gene pairs remained similar (~20,000) regardless of the direction of the observed correlation (Figure 7f, top). Furthermore, while the number of unique dmCpG‐gene pairs in the aging context was much reduced (~3,000), the proportion compared to the total number of strong correlations observed in a given dataset remained, to a great extent, similar (Figure 7f, bottom). Interestingly, the differences between the genomic distributions of the unique hyper‐ and hypomethylated dmCpG‐gene pairs identified in aging or in cancer followed the same trend to those observed for the genomic distribution of the hyper‐ and hypomethylated dmCpGs identified in each of these processes (Figure 2b,c), with hypermethylated dmCpGs being more enriched in CpG islands in aging as compared to the array (Fisher's tests; both *p *<* *.001, ORs = 2.0, 1.4, for positively and negatively correlated dmCpGs, respectively), in contrast to the enrichment at open sea locations (Fisher's tests; all *p *<* *.001, ORs = 4.6, 4.1, 2.3 and 2.5 for positively and negatively correlated dmCpGs in aging and cancer, respectively) and intronic regions (Fisher's tests *p *<* *.002, <.03, <.001 and <.001, ORs = 2.2, 1.9, 1.6, and 1.7 for positively and negatively correlated dmCpGs in aging and cancer, respectively) of the hypomethylated dmCpGs in both processes (Figure 7g,h). It is worth noting that the distribution of the unique hypermethylated dmCpGs which also control gene expression was more enriched in open sea locations as compared to dmCpGs in general in both aging and cancer (Figure 7g as compared to Figure 2b).

Finally, we compared the unique dmCpGs that displayed strong correlations between DNA methylation and gene expression in aging or in cancer (Figure 7i). We observed an extensive overlap between probes that displayed positive or negative correlations in the two processes (Fisher's tests, all *p *<* *.001, ORs = 908, 2244, 205, and 171; JIs = 0.57, 0.54, 0.57, and 0.54 for aging and cancer hyper‐ and hypomethylated CpGs, respectively). This fact might explain their similar genomic distributions (Figure 7g,h), indicating that most of these dmCpGs could play a dual role in the control of their different gene expression targets. We also observed a considerable overlap between dmCpGs associated with gene expression identified in aging and cancer processes (Fisher's tests, both *p *<* *.001, ORs = 5.1 and 9.1; JIs = 0.06 and 0.03, for hyper‐ and hypomethylated CpGs, respectively). Interestingly, regardless of whether the DNA methylation change was toward hyper‐ or hypomethylation, ~ 60–70% of the aging‐related dmCpGs which controlled gene expression were also present in the group of cancer‐related dmCpGs (Figure 7i). These results point toward similarities of cancer‐ and aging‐related dmCpGs in the control of gene expression in normal tissue, despite the fact that the number of cancer‐related dmCpGs is clearly larger than their aging counterparts.

## 3 DISCUSSION

Although it is widely accepted that cancer is an age‐dependent disease, the underlying molecular mechanisms are still poorly characterized. In this work, we have looked at the similarities and differences in epigenetic changes associated with cancer and aging.

In agreement with previously published data (Fernández et al., 2015), we observed that the number of aging‐ and cancer‐associated DNA methylation changes was variable and, in the case of aging, had a marked tissue type‐dependent component. In general, cancer displayed strong and bidirectional changes, while, strikingly, hypermethylated CpG sites were predominantly observed for the aging process. These results, which are ostensibly in contrast with the classically described global hypomethylation changes in cancer and aging, might potentially arise from the limitations of our study. As the methylation arrays used in our analyses mainly interrogate genetic elements and do not include repeated DNA, which covers a substantial fraction of the genome and frequently loses DNA methylation in tumors and aged cells, the genome‐wide landscape may be different (Ehrlich, 2009). Nonetheless, epigenetic signatures have been successfully derived previously using array technology (Fernández et al., 2015; Rakyan et al., 2010; Teschendorff et al., 2010) and our results are in line with recent studies which report no global decreases in DNA hypomethylation with aging in diverse mouse tissues, such as liver (Cole et al., 2017; Hahn et al., 2017), hippocampus (Masser et al., 2017), or hematopoietic stem cells (Beerman et al., 2013; Sun et al., 2014), thus strengthening the validity of our observations.

The changes in cell type composition that occur with age and cancer are also well‐known confounding factors that could affect our datasets (Zheng et al., 2017). However, the application of the SVA method of correction (and Houseman correction for blood) and the use of a pure‐cell dataset such as the glia dataset (Guintivano et al., 2013) allowed us to tackle this issue in two different ways. Additionally, the use of the blood validation dataset (Hannum et al., 2013) allowed us to verify the reliability of our workflow, as in terms of whole blood dmCpGs we obtained 89% concordance with previous studies using the same data (Fernández et al., 2015).

When analyzing the genomic distribution of dmCpGs and, in line with previously published reports (Cruickshanks et al., 2013; Kulis et al., 2012; Yuan et al., 2015), we found that hypomethylated CpGs were enriched at open sea DNA regions, principally intronic and intergenic, irrespective of the type of process. The distribution of hypermethylated CpGs was found to be similar to that of the array, which is to a certain extent to be expected because it was designed to interrogate a promoter‐ and CpG dense‐biased portion of the genome. Nonetheless, hypermethylation changes always occurred in far more CpG‐dense regions than hypomethylation changes (Day et al., 2013; Yuan et al., 2015), and this observed effect was especially noticeable for aging dmCpGs.

When studying the potential effect of tissue type on DNA methylation changes, we found, in agreement with recently published data (Chen, Breeze, Zhen, Beck & Teschendorff, 2016), that DNA methylation changes in different tumor types were surprisingly similar, regardless of the tendency of the alteration. This observation is conceptually relevant because it has classically been considered that different tumor types are characterized by specific DNA methylation signatures (Ehrlich & Jiang, 2005; Portela & Esteller, 2010). In this sense, our data confirm that, although different tumor types might display specific DNA methylation patterns, there is a significant common nexus between them. The analysis of the DNA methylation changes observed with respect to the aging process also revealed a significant overlap between tissue types, although it is possible that these results are affected by the variability in the sizes of the sets of probes detected in the aging analysis.

Our data revealed that dmCpGs shared by two or more tissues were much less likely to have different behaviors in other tissues, perhaps pointing toward nonstochastic and possibly functional roles for these CpGs.

The systematic DNA methylation analyses described in this study confirm that DNA hypermethylation in aging and cancer is associated with the same set of histone marks, including the repressive H3K27me3 and H3K9me3 marks, and the activating H3K4me1/3 posttranslational modifications. Chromatin state analysis revealed that the hypermethylation‐associated H3K27me3 and H3K4me1/3 marks configured bivalent chromatin domains, as has been extensively described in embryonic stem cells (Fernández et al., 2015; Ohm et al., 2007; Rakyan et al., 2010; Schlesinger et al., 2007; Teschendorff et al., 2010; Widschwendter et al., 2007). Moreover, our data reveal that this chromatin signature is not restricted to only embryonic stem cells, but rather this trend should be considered an extended, global tissue‐independent chromatin signature of DNA hypermethylation in aging and cancer. Interestingly, Chen and colleagues (Chen et al., 2016) have recently demonstrated that normal tissue signatures are better predictors of DNA hypermethylation changes than ESC signatures. Furthermore, hypermethylation changes were also associated with the repressive histone mark H3K9me3 (Ohm et al., 2007), which was correlated to ZNF genes and DNA repeats in our chromatin state analyses, and which might have a potential relationship with the malignant transformation process (Severson, Tokar, Vrba, Waalkes & Futscher, 2013).

Regarding DNA hypomethylation, our results showed that age‐related DNA hypomethylation is associated with the activating histone posttranslational modification H3K4me1, which supports previously published data (Fernández et al., 2015). A slight tendency for the enrichment of H3K27Ac, a histone mark characteristic of active enhancers (Creyghton et al., 2010), was also detected in our analyses. Intriguingly, the chromatin signature of DNA hypomethylation in cancer was substantially different, being primarily enriched in the posttranslational repressive histone modification H3K9me3, a relationship that has been investigated in colon and breast cancer (Berman et al., 2011; Hon et al., 2012). This observation might be conceptually relevant because DNA methylation has been proposed to be a molecular link between aging and cancer (Fraga, Agrelo & Esteller, 2007; Klutstein, Nejman, Greenfield & Cedar, 2016). However, our results suggest that the role of DNA methylation as a possible link between aging and cancer is more complex than previously proposed. Importantly, even though many of the observed DNA methylation changes in aging were not shared by tissues, we were able to describe a common chromatin signature characteristic of the aging process.

Regarding the analysis of the chromatin states, DNA hypomethylation in cancer was associated with heterochromatin DNA regions, which is in line with previous work (Berman et al., 2011; Kulis et al., 2012). In contrast, chromatin marks of DNA hypomethylation in aging were associated with enhancers, reinforcing previous observations performed with the Infinium HumanMethylation27K Beadchip platform (Day et al., 2013). As DNA methylation changes in enhancers have been shown to play an important role in gene regulation (Aran, Sabato & Hellman, 2013; Blattler et al., 2014; Heyn et al., 2016), our results suggest that DNA hypomethylation during aging might have a different functional role in gene regulation compared to DNA hypomethylation changes in cancer.

With regard to the potential effectors of the distinct chromatin signatures, enrichment analyses of transcription factors revealed the presence of EZH2 and SUZ12 polycomb components at DNA hypermethylated sites, both in cancer and aging. Specific aging hypermethylation‐associated factors were also observed in our comparisons, such as REST, which has been reported in previously published data in blood (Yuan et al., 2015), and has also been correlated with longevity (Lu et al., 2014). Concerning DNA hypomethylation, transcription factors such as FOS, JUN, and JUND were detected at both cancer and aging hypomethylated CpG sites, but again aging displayed stronger and more varied enrichment, and included the presence FOSL1/2, other bZIP‐domain factors like MAFF and MAFK, and STAT3, which has been associated with recruitment of the H3K4 methyltransferase SET9 at promoters (Yang et al., 2010). Altogether, these observations would imply that hypomethylation in aging displays a more marked functional context than that of cancer, exhibiting an increased enrichment of some factors also detected at cancer hypomethylated sites and other specific factors not found associated with tumoral changes.

Interestingly, our gene ontology analyses revealed similar gene functionalities affected by cancer and aging DNA hypermethylation, mainly related to developmental processes, which is in line with the methylation of bivalent chromatin promoters of developmental regulators in cancer and aging (Easwaran et al., 2012; Rakyan et al., 2010). On the other hand, DNA hypomethylation in cancer was mainly associated with functions identified with cellular signaling, and much lower enrichments in gene functions were found for hypomethylated CpGs in aging. A preponderance of nongenic enhancer hypomethylation in aging could potentially explain this absence of gene function association in our data.

To date, the potential relationships between DNA methylation and gene expression have only been systematically analyzed in a small subset of studies (Gevaert, Tibshirani & Plevritis, 2015; Gutierrez‐Arcelus et al., 2013, 2015), and the potential effects of these relationships on aging and cancer are yet to be elucidated. To this end, we explored the establishment of potential correlations between these two processes using the TCGA‐KIRC dataset. While most correlative studies focus on CpGs located at particular genomic regions, such as DNA promoters (Moarii, Boeva, Vert & Reyal, 2015) and cis‐related correlations with the gene of interest (Gutierrez‐Arcelus et al., 2015), we performed a nonbiased approach focusing on all the potential pairwise comparisons that could be identified between any significant dmCpG and the genes expressed in the context of normal kidney tissue. The limitations of these analyses did not allow us to distinguish between direct (i.e., mediated by the effects of the DNA methylation process itself) or indirect regulation of gene expression governed by the subsequent expression of other regulatory factors. Nonetheless, we observed that both aging and cancer dmCpGs influence gene expression to a similar extent, as these processes show the same proportions of strong correlations between DNA methylation and gene expression in normal kidney tissue. Moreover, we observed a similar number of positive and negative correlations between DNA methylation and gene expression, as described in Gutierrez‐Arcelus et al., 2013, with most of these positively and negatively correlated dmCpGs overlapping substantially, suggesting that these CpG sites may play a dual role in the control of gene expression, or the involvement of other factors.

Finally, we found that most of the tumor types analyzed in this study did not show age‐associated DNA methylation changes, which is in agreement with the reprogramming of the epigenetic clock in cancer cells (Horvath, 2013). As an exception, we identified age‐associated dmCpGs in thyroid tumors. Uncommonly, thyroid cancer includes age as a prognostic indicator in most staging systems (Haymart, 2009), implying that these cancers do suffer age‐related changes in their behavior. Intriguingly, this tissue displayed the lowest level of DNA methylation changes in cancer and one of the lowest in aging. Although the reasons for the different behavior of DNA methylation changes in thyroid are currently unknown, they could be related to the good prognosis that typically characterizes this type of tumor. In fact, Yang Z. and collaborator's “epiTOC” mitotic clock (Yang et al., 2016) shows thyroid cancer to have the least deviation from the behavior of its normal tissue. In this regard, future research should be conducted to address this issue.

In conclusion, our results indicate that hyper‐ and hypomethylated changes in aging and cancer each have similar genomic distributions and manifest tissue‐independent trends in both processes. We confirm that chromatin signatures of DNA hypermethylation in aging and cancer are similar but, strikingly, we demonstrate that they are different for DNA hypomethylation. Collectively, our data suggest that the possible role of DNA methylation as a molecular link between aging and cancer is more complex than previously thought.

## 4 EXPERIMENTAL PROCEDURES

### 4.1 Data acquisition

HumanMethylation450 BeadChip (Illumina, California, USA) DNA methylation data (Level 3) corresponding to normal or primary tumors from breast (BRCA), kidney (KIRC), thyroid (THCA), skin (SKCM), and glia (GBM) samples were obtained from TCGA consortium via UCSC Xena Public Data Hub (http://xena.ucsc.edu/). Kidney, skin, and glia tissue datasets were enlarged for control cases using additional samples from KIRP (TCGA), skin (Bormann et al., 2016), and glia (Guintivano et al., 2013), respectively. Tissues were chosen based on disease prevalence, control data availability, and previous literature analyses to include both novel and pre‐analyzed tissues. We also performed analyses on two supplementary datasets: lung adenocarcinoma (LUAD) and control TCGA data, and whole blood from a healthy cohort (Hannum et al., 2013). Extended information about the samples for each tissue type is shown in Tables 1, S1 and S9. Data were preprocessed as detailed in supporting information.

### 4.2 Differential DNA methylation analyses

Differentially methylated probes (dmCpGs) in aging and cancer were calculated with the R package *limma* (version 3.32.2) (Ritchie et al., 2015). Briefly, a linear model between methylation levels as response variable, the variable of interest (either *age* group or *sample_type*), and surrogate variables (see Supplementary Methods) was fitted for each of the analyses, adjusting *p*‐values to control for false discovery rate (FDR < 0.05). For the calculation of age‐related dmCpGs, samples were divided into age quantiles in such a way as to obtain groups with sizes of *n* = 15–30, and comparisons were performed between the upper (*OLD*) and the lower (*YOUNG*) quantile. Cancer‐related dmCpGs were calculated between normal tissue (*Solid Tissue Normal*) and tumor samples (*Primary Tumor*) as indicated in Tables 1, S1 and S2. Probes with M‐value changes of <0.5 were not considered as dmCpGs, as has been suggested elsewhere (Du et al., 2010). Venn diagrams of relationships between dmCpGs were generated with the online resource provided by the UGent/VIB bioinformatics unit (http://bioinformatics.psb.ugent.be/webtools/Venn/). Further enrichment analyses were performed by means of two‐sided Fisher's tests (*p *<* *.05 significance threshold), measuring effect size either by odds ratios (OR), or by the difference between observed counts and expected hypergeometric mean (EHM), employing appropriate backgrounds of interrogated probes for the given context.

Density of CpG (related to Figure 2a), CGI status and genomic region (related to Figure 2b,c), and analysis, DNA methylation age (related to Figure 1d), and gene and KEGG ontology (related to Figure 6a) analyses are further detailed in Supplementary Methods.

### 4.3 Region set enrichment analysis

Enrichment analyses were performed with the R package *LOLA* (version 1.4.0) (Sheffield & Bock, 2016), which looks for over‐enrichment by conducting one‐sided Fisher's tests (*p *<* *.05 significance threshold), by comparing overlap of probes (10 bp probe‐centered windows) with the dataset of interest. Enrichment of histone marks was determined using histone ChIP‐seq peak tracks (H3K4me1, H3K4me3, H3K27me3, H3K36me3, H3K9me3, and H3K27ac marks) from 98 epigenomes (primary tissues, cultures, and cell lines) obtained from the NIH Roadmap and ENCODE projects (Bernstein et al., 2010; Consortium 2012) (datasets obtained from http://databio.org/regiondb) (see Table S8). The same method was employed for chromatin‐segment analysis using NIH Roadmap's ChromHMM expanded 18‐state model tracks for the same 98 epigenomes (see Figure S7 and Table S10, custom database generated with data obtained from http://egg2.wustl.edu/roadmap/). In a similar fashion, ChIP‐seq peak tracks from ENCODE for transcription factor binding sites (TFBS) comprising 689 datasets corresponding to 188 TFs analyzed in 91 cell and tissue types were employed for TFBS enrichment analysis (http://databio.org/regiondb, see Table S11).

### 4.4 Gene expression analyses

Gene expression data corresponding to RNAseq HTSeq‐Counts from the GDC TCGA Kidney Clear Cell Carcinoma (KIRC) cohort were obtained from UCSC Xena Public Data Hub (http://xena.ucsc.edu/, dataset ID: TCGA‐KIRC/Xena_Matrices/TCGA‐KIRC.htseq_counts.tsv). Samples were filtered to fulfill the criteria of using only those cases with paired DNA methylation and gene expression data. Log2(count+1) data were further transformed to obtain integer count reads per gene condition. Nonvariable and low‐expressed genes (sum of expression across all the samples <1,000 counts) were removed to reduce the number of noninformative conditions. Differential expression analyses were performed with the R package DESeq2 (version 1.16.1) (Love, Huber & Anders, 2014), using the standard workflow and parameters, defining differentially expressed genes if they satisfied *p *<* *.05 after adjustment for multiple testing. For gene expression and DNA methylation correlation analyses, RNAseqV2 log2(RSEM+1) normalized level 3 TCGA gene expression data were obtained for kidney normal tissue (KIRC) via UCSC Xena Public Data Hub (http://xena.ucsc.edu/). Samples were filtered so as to use only those with paired DNA methylation data. Nonvariable and low‐expression genes (those with the sum of expression between all the samples of <10) were discarded. After filtering, pairwise Spearman correlations between DNA methylation level and gene expression level were calculated for all the combinations of probes and genes in normal kidney tissue samples, using probes that were previously detected dmCpGs in cancer and aging.

### 4.5 Availability

All data generated during this study are included in this published article and its supplementary information files and are also available in the Zenodo public repository, https://doi.org/10.5281/zenodo.1086491.

## CONFLICT OF INTEREST

None declared.

## AUTHORS’ CONTRIBUTION

M.F.F., A.F.F., and G.F.B. conceived, coordinated, and supervised the study. M.F.F, R.F.P., and J.R.T. designed all aspects of the research and contributed equally to this work. R.F.P. and J.R.T. collected the data and performed computational analyses. M.F.F., A.F.F., R.F.P., and J.R.T. wrote the manuscript. All authors revised, read, and approved the final manuscript.

## References

[acel12744-bib-0001] Aran, D. , Sabato, S. , & Hellman, A. (2013). DNA methylation of distal regulatory sites characterizes dysregulation of cancer genes. Genome Biology, 14, R21 10.1186/gb-2013-14-3-r21 23497655 PMC4053839

[acel12744-bib-0002] Beerman, I. , Bock, C. , Garrison, B. S. , Smith, Z. D. , Gu, H. , Meissner, A. , & Rossi, D. J. (2013). Proliferation‐dependent alterations of the DNA methylation landscape underlie hematopoietic stem cell aging. Cell Stem Cell, 12, 413–425. 10.1016/j.stem.2013.01.017 23415915 PMC12163706

[acel12744-bib-0003] Berman, B. P. , Weisenberger, D. J. , Aman, J. F. , Hinoue, T. , Ramjan, Z. , Liu, Y. , … Laird, P. W. (2011). Regions of focal DNA hypermethylation and long‐range hypomethylation in colorectal cancer coincide with nuclear lamina–associated domains. Nature Genetics, 44, 40–46. 22120008 10.1038/ng.969 PMC4309644

[acel12744-bib-0004] Bernstein, B. E. , Stamatoyannopoulos, J. A. , Costello, J. F. , Ren, B. , Milosavljevic, A. , Meissner, A. , … Thomson, J. A. (2010). The NIH roadmap epigenomics mapping consortium. Nature Biotechnology, 28, 1045–1048. 10.1038/nbt1010-1045 PMC3607281 20944595

[acel12744-bib-0005] Blattler, A. , Yao, L. , Witt, H. , Guo, Y. , Nicolet, C. M. , Berman, B. P. , & Farnham, P. J. (2014). Global loss of DNA methylation uncovers intronic enhancers in genes showing expression changes. Genome Biology, 15, 469 10.1186/s13059-014-0469-0 25239471 PMC4203885

[acel12744-bib-0006] Bormann, F. , Rodríguez‐Paredes, M. , Hagemann, S. , Manchanda, H. , Kristof, B. , Gutekunst, J. , … Lyko, F. (2016). Reduced DNA methylation patterning and transcriptional connectivity define human skin aging. Aging Cell, 15, 563–571. 10.1111/acel.12470 27004597 PMC4854925

[acel12744-bib-0007] Chen, Y. , Breeze, C. E. , Zhen, S. , Beck, S. , & Teschendorff, A. E. (2016). Tissue‐independent and tissue‐specific patterns of DNA methylation alteration in cancer. Epigenetics Chromatin, 9, 10 Retrieved from http://www.epigeneticsandchromatin.com/content/9/1/10 26958079 10.1186/s13072-016-0058-4 PMC4782576

[acel12744-bib-0008] Cole, J. J. , Robertson, N. A. , Rather, M. I. , Thomson, J. P. , McBryan, T. , Sproul, D. , … Adams, P. D. (2017). Diverse interventions that extend mouse lifespan suppress shared age‐associated epigenetic changes at critical gene regulatory regions. Genome Biology, 18, 58 10.1186/s13059-017-1185-3 28351383 PMC5370462

[acel12744-bib-0009] Consortium TEP (2012). An integrated encyclopedia of DNA elements in the human genome. Nature, 489, 57–74. 22955616 10.1038/nature11247 PMC3439153

[acel12744-bib-0010] Creyghton, M. P. , Cheng, A. W. , Welstead, G. G. , Kooistra, T. , Carey, B. W. , Steine, E. J. , … Jaenisch, R. (2010). Histone H3K27ac separates active from poised enhancers and predicts developmental state. Proceedings of the National Academy of Sciences of the United States of America, 107, 21931–21936. 10.1073/pnas.1016071107 21106759 PMC3003124

[acel12744-bib-0011] Cruickshanks, H. A. , McBryan, T. , Nelson, D. M. , VanderKraats, N. D. , Shah, P. P. , van Tuyn, J. , … Adams, P. D. (2013). Senescent cells harbour features of the cancer epigenome. Nature Cell Biology, 15, 1495–1506. 10.1038/ncb2879 24270890 PMC4106249

[acel12744-bib-0012] Day, K. , Waite, L. L. , Thalacker‐Mercer, A. , West, A. , Bamman, M. M. , Brooks, J. D. , … Absher, D. (2013). Differential DNA methylation with age displays both common and dynamic features across human tissues that are influenced by CpG landscape. Genome Biology, 14, R102 10.1186/gb-2013-14-9-r102 24034465 PMC4053985

[acel12744-bib-0013] de Magalhães, J. P. (2013). How ageing processes influence cancer. Nature Reviews Cancer, 13, 357–365. 10.1038/nrc3497 23612461

[acel12744-bib-0014] DePinho, R. A. (2000). The age of cancer. Nature, 408, 248–254. 10.1038/35041694 11089982

[acel12744-bib-0015] Du, P. , Zhang, X. , Huang, C.‐C. , Jafari, N. , Kibbe, W. A. , Hou, L. , & Lin, S. M. (2010). Comparison of Beta‐value and M‐value methods for quantifying methylation levels by microarray analysis. BMC Bioinformatics, 11, 587 10.1186/1471-2105-11-587 21118553 PMC3012676

[acel12744-bib-0016] Easwaran, H. , Johnstone, S. E. , Van Neste, L. , Ohm, J. , Mosbruger, T. , Wang, Q. , … Baylin, S. B. (2012). A DNA hypermethylation module for the stem/progenitor cell signature of cancer. Genome Research, 22, 837–849. 10.1101/gr.131169.111 22391556 PMC3337430

[acel12744-bib-0017] Ehrlich, M. (2009). DNA hypomethylation in cancer cells. Epigenomics, 1, 239–259. 10.2217/epi.09.33 20495664 PMC2873040

[acel12744-bib-0018] Ehrlich, M. , Jiang, G. (2005). DNA Hypo‐ vs. Hypermethylation in Cancer In: DNA Methylation and Cancer Therapy. Medical Intelligence Unit. Springer, Boston. MA pp. 31–41. Retrieved from http://link.springer.com/chapter/10.1007/0-387-27443-X_3

[acel12744-bib-0019] Fernández, A. F. , Bayón, G. F. , Urdinguio, R. G. , Toraño, E. G. , García, M. G. , Carella, A. , … Fraga, M. F. (2015). H3K4me1 marks DNA regions hypomethylated during aging in human stem and differentiated cells. Genome Research, 25, 27–40. 10.1101/gr.169011.113 25271306 PMC4317171

[acel12744-bib-0020] Fraga, M. F. , Agrelo, R. , & Esteller, M. (2007). Cross‐Talk between aging and cancer: The epigenetic language. Annals of the New York Academy of Sciences, 1100, 60–74. 10.1196/annals.1395.005 17460165

[acel12744-bib-0021] Fraga, M. F. , & Esteller, M. (2007). Epigenetics and aging: The targets and the marks. Trends in Genetics, 23, 413–418. 10.1016/j.tig.2007.05.008 17559965

[acel12744-bib-0022] Gevaert, O. , Tibshirani, R. , & Plevritis, S. K. (2015). Pancancer analysis of DNA methylation‐driven genes using MethylMix. Genome Biology, 16, 17 10.1186/s13059-014-0579-8 25631659 PMC4365533

[acel12744-bib-0023] Guintivano, J. , Aryee, M. J. , & Kaminsky, Z. A. (2013). A cell epigenotype specific model for the correction of brain cellular heterogeneity bias and its application to age, brain region and major depression. Epigenetics, 8, 290–302. 10.4161/epi.23924 23426267 PMC3669121

[acel12744-bib-0024] Gutierrez‐Arcelus, M. , Lappalainen, T. , Montgomery, S. B. , Buil, A. , Ongen, H. , Yurovsky, A. , … Dermitzakis, E. T. (2013). Passive and active DNA methylation and the interplay with genetic variation in gene regulation. eLife, 2, e00523. 23755361 10.7554/eLife.00523 PMC3673336

[acel12744-bib-0025] Gutierrez‐Arcelus, M. , Ongen, H. , Lappalainen, T. , Montgomery, S. B. , Buil, A. , Yurovsky, A. , … Dermitzakis, E. T. (2015). Tissue‐specific effects of genetic and epigenetic variation on gene regulation and splicing. PLoS Genetics, 11, e1004958 10.1371/journal.pgen.1004958 25634236 PMC4310612

[acel12744-bib-0026] Hahn, O. , Grönke, S. , Stubbs, T. M. , Ficz, G. , Hendrich, O. , Krueger, F. , … Partridge, L. (2017). Dietary restriction protects from age‐associated DNA methylation and induces epigenetic reprogramming of lipid metabolism. Genome Biology, 18, 56 10.1186/s13059-017-1187-1 28351387 PMC5370449

[acel12744-bib-0027] Hannum, G. , Guinney, J. , Zhao, L. , Zhang, L. , Hughes, G. , Sadda, S. , … Zhang, K. (2013). Genome‐wide methylation profiles reveal quantitative views of human aging rates. Molecular Cell, 49, 359–367. 10.1016/j.molcel.2012.10.016 23177740 PMC3780611

[acel12744-bib-0028] Haymart, M. R. (2009). Understanding the relationship between age and thyroid cancer. The Oncologist, 14, 216–221. 10.1634/theoncologist.2008-0194 19270027

[acel12744-bib-0029] Heyn, H. , Li, N. , Ferreira, H. J. , Moran, S. , Pisano, D. G. , Gomez, A. , … Esteller, M. (2012). Distinct DNA methylomes of newborns and centenarians. Proceedings of the National Academy of Sciences of the United States of America, 109, 10522–10527. 10.1073/pnas.1120658109 22689993 PMC3387108

[acel12744-bib-0030] Heyn, H. , Vidal, E. , Ferreira, H. J. , Vizoso, M. , Sayols, S. , Gomez, A. , … Esteller, M. (2016). Epigenomic analysis detects aberrant super‐enhancer DNA methylation in human cancer. Genome Biology, 17, 11 10.1186/s13059-016-0879-2 26813288 PMC4728783

[acel12744-bib-0031] Hon, G. C. , Hawkins, R. D. , Caballero, O. L. , Lo, C. , Lister, R. , Pelizzola, M. , … Ren, B. (2012). Global DNA hypomethylation coupled to repressive chromatin domain formation and gene silencing in breast cancer. Genome Research, 22, 246–258. 10.1101/gr.125872.111 22156296 PMC3266032

[acel12744-bib-0032] Horvath, S. (2013). DNA methylation age of human tissues and cell types. Genome Biology, 14, R115 10.1186/gb-2013-14-10-r115 24138928 PMC4015143

[acel12744-bib-0033] Klutstein, M. , Nejman, D. , Greenfield, R. , & Cedar, H. (2016). DNA methylation in cancer and aging. Cancer Research, 76, 3446–3450. 10.1158/0008-5472.CAN-15-3278 27256564

[acel12744-bib-0034] Kulis, M. , Heath, S. , Bibikova, M. , Queirós, A. C. , Navarro, A. , Clot, G. , … Martín‐Subero, J. I. (2012). Epigenomic analysis detects widespread gene‐body DNA hypomethylation in chronic lymphocytic leukemia. Nature Genetics, 44, 1236–1242. 10.1038/ng.2443 23064414

[acel12744-bib-0035] Love, M. I. , Huber, W. , & Anders, S. (2014). Moderated estimation of fold change and dispersion for RNA‐seq data with DESeq2. Genome Biology, 15, 550 10.1186/s13059-014-0550-8 25516281 PMC4302049

[acel12744-bib-0036] Lu, T. , Aron, L. , Zullo, J. , Pan, Y. , Kim, H. , Chen, Y. , … Yankner, B. A. (2014). REST and stress resistance in ageing and Alzheimer's disease. Nature, 507, 448–454. 10.1038/nature13163 24670762 PMC4110979

[acel12744-bib-0037] Masser, D. R. , Hadad, N. , Porter, H. L. , Mangold, C. A. , Unnikrishnan, A. , Ford, M. M. , … Freeman, W. M. (2017). Sexually divergent DNA methylation patterns with hippocampal aging. Aging Cell, 16, 1342–1352. 10.1111/acel.12681 28948711 PMC5676057

[acel12744-bib-0038] Moarii, M. , Boeva, V. , Vert, J.‐P. , & Reyal, F. (2015). Changes in correlation between promoter methylation and gene expression in cancer. BMC Genomics, 16, 873 10.1186/s12864-015-1994-2 26510534 PMC4625954

[acel12744-bib-0039] Ohm, J. E. , McGarvey, K. M. , Yu, X. , Cheng, L. , Schuebel, K. E. , Cope, L. , … Baylin, S. B. (2007). A stem cell‐like chromatin pattern may predispose tumor suppressor genes to DNA hypermethylation and silencing in adult cancers. Nature Genetics, 39, 237–242. 10.1038/ng1972 17211412 PMC2744394

[acel12744-bib-0040] Portela, A. , & Esteller, M. (2010). Epigenetic modifications and human disease. Nature Biotechnology, 28, 1057–1068. 10.1038/nbt.1685 20944598

[acel12744-bib-0041] Rakyan, V. K. , Down, T. A. , Maslau, S. , Andrew, T. , Yang, T.‐P. , Beyan, H. , … Spector, T. D. (2010). Human aging‐associated DNA hypermethylation occurs preferentially at bivalent chromatin domains. Genome Research, 20, 434–439. 10.1101/gr.103101.109 20219945 PMC2847746

[acel12744-bib-0042] Ritchie, M. E. , Phipson, B. , Wu, D. , Hu, Y. , Law, C. W. , Shi, W. , & Smyth, G. K. (2015). Limma powers differential expression analyses for RNA‐sequencing and microarray studies. Nucleic Acids Research, 43, e47 10.1093/nar/gkv007 25605792 PMC4402510

[acel12744-bib-0043] Rodríguez‐Paredes, M. , & Esteller, M. (2011). Cancer epigenetics reaches mainstream oncology. Nature Medicine, 17, 330–339. 10.1038/nm.2305 21386836

[acel12744-bib-0044] Schlesinger, Y. , Straussman, R. , Keshet, I. , Farkash, S. , Hecht, M. , Zimmerman, J. , … Cedar, H. (2007). Polycomb‐mediated methylation on Lys27 of histone H3 pre‐marks genes for de novo methylation in cancer. Nature Genetics, 39, 232–236. 10.1038/ng1950 17200670

[acel12744-bib-0045] Severson, P. L. , Tokar, E. J. , Vrba, L. , Waalkes, M. P. , & Futscher, B. W. (2013). Coordinate H3K9 and DNA methylation silencing of ZNFs in toxicant‐induced malignant transformation. Epigenetics, 8, 1080–1088. 10.4161/epi.25926 23974009 PMC3891689

[acel12744-bib-0046] Sheffield, N. C. , & Bock, C. (2016). LOLA: Enrichment analysis for genomic region sets and regulatory elements in R and bioconductor. Bioinformatics, 32, 587–589. 10.1093/bioinformatics/btv612 26508757 PMC4743627

[acel12744-bib-0047] Sun, D. , Luo, M. , Jeong, M. , Rodriguez, B. , Xia, Z. , Hannah, R. , … Goodell, M. A. (2014). Epigenomic profiling of young and aged HSCs reveals concerted changes during aging that reinforce self‐renewal. Cell Stem Cell, 14, 673–688. 10.1016/j.stem.2014.03.002 24792119 PMC4070311

[acel12744-bib-0048] Teschendorff, A. E. , Menon, U. , Gentry‐Maharaj, A. , Ramus, S. J. , Weisenberger, D. J. , Shen, H. , … Widschwendter, M. (2010). Age‐dependent DNA methylation of genes that are suppressed in stem cells is a hallmark of cancer. Genome Research, 20, 440–446. 10.1101/gr.103606.109 20219944 PMC2847747

[acel12744-bib-0049] Widschwendter, M. , Fiegl, H. , Egle, D. , Mueller‐Holzner, E. , Spizzo, G. , Marth, C. , … Laird, P. W. (2007). Epigenetic stem cell signature in cancer. Nature Genetics, 39, 157–158. 10.1038/ng1941 17200673

[acel12744-bib-0050] Yang, J. , Huang, J. , Dasgupta, M. , Sears, N. , Miyagi, M. , Wang, B. , … Stark, G. R. (2010). Reversible methylation of promoter‐bound STAT3 by histone‐modifying enzymes. Proceedings of the National Academy of Sciences of the United States of America, 107, 21499–21504. 10.1073/pnas.1016147107 21098664 PMC3003019

[acel12744-bib-0051] Yang, Z. , Wong, A. , Kuh, D. , Paul, D. S. , Rakyan, V. K. , Leslie, R. D. , … Teschendorff, A. E. (2016). Correlation of an epigenetic mitotic clock with cancer risk. Genome Biology, 17, 205 10.1186/s13059-016-1064-3 27716309 PMC5046977

[acel12744-bib-0052] Yuan, T. , Jiao, Y. , de Jong, S. , Ophoff, R. A. , Beck, S. , & Teschendorff, A. E. (2015). An integrative multi‐scale analysis of the dynamic DNA methylation landscape in aging. PLoS Genetics, 11, e1004996 10.1371/journal.pgen.1004996 25692570 PMC4334892

[acel12744-bib-0053] Zheng, S. C. , Beck, S. , Jaffe, A. E. , Koestler, D. C. , Hansen, K. D. , Houseman, A. E. , … Teschendorff, A. E. (2017). Correcting for cell‐type heterogeneity in epigenome‐wide association studies: Revisiting previous analyses. Nature Methods, 14, 216–217. 10.1038/nmeth.4187 28245219 PMC6659733

[acel12744-bib-0054] Zykovich, A. , Hubbard, A. , Flynn, J. M. , Tarnopolsky, M. , Fraga, M. F. , Kerksick, C. , … Melov, S. (2014). Genome‐wide DNA methylation changes with age in disease‐free human skeletal muscle. Aging Cell, 13, 360–366. 10.1111/acel.12180 24304487 PMC3954952

