# Supplementary material for: Distinct chromatin signatures of DNA hypomethylation in aging and cancer
Source: Aging Cell. 2018 Mar 5;17(3):e12744. doi: 10.1111/acel.12744 (PMC5946083; doi:10.1111/acel.12744)
Supplement: Supplementary file 1 [file ACEL-17-e12744-s001.pdf]

SUPPLEMENTARY METHODS

Data preprocessing. HumanMethylation450 BeadChip files containing β-values for ~450K probes had already been preprocessed and released by TCGA according to Data Level 3 guidelines. Probes located in chromosomes X and Y, probes overlapping genetic variants (SNP137Common track from UCSC genome browser), crossreactive and multimapping probes (Chen et al. 2013), as well as probes masked as NA ('Not Available') were discarded for downstream analyses. For each of the datasets, all samples (normal and tumor cases) were normalized using the BMIQ method (Teschendorff et al. 2013) implemented in the R/Bioconductor package *ChAMP* (version 2.8.1)(Morris et al. 2014). In the case of skin and glia tissues, since controls were obtained from various different studies, we performed a prior quantile normalization on these datasets (Touleimat & Tost 2012), as implemented in the R/Bioconductor package *wateRmelon* (version 1.18.0)(Pidsley et al. 2013). In the case of blood tissue, a specific cell-heterogeneity correction was applied as described by (Houseman et al. 2012). M-values were calculated from the normalized β-values through a logit transformation (R/Bioconductor package *lumi*, version 2.28.0)(Du et al. 2008) and employed for downstream statistical analyses. Multidimensional scaling (MDS) and principal component regression analyses were used to identify potential confounding variables (R/Bioconductor package *Enmix*, version 1.12.3)(Xu et al. 2016). To further correct for batch effects and unwanted sources of variation in the data, we performed a surrogate variable analysis (Leek & Storey 2007) with the R/Bioconductor package *sva* (version 3.24.0). Surrogate variables for the different tissue datasets corresponding to "aging" (control cases or primary tumors) or "cancer" (control cases versus primary tumors) analyses were calculated employing the variable *age* or *sample_type* as the outcome of interest respectively. The number of latent factors for each of the different datasets was estimated using the "*leek*" method.

Density of CpG analysis. For each of the probes in the HumanMethylation450 microarray, density of CpG was measured as the number of CpGs present divided by the number of those possible in a 2 kbp window centered on the CpG under study. Wilcoxon non-parametric tests were used to determine whether there were significant difference between the density distributions of the CpGs belonging to each subset of interest and the densities of the array probes in the background. A significance level of 0.05 was employed for all tests. Shift size was measured using median differences and Cliff's Delta (D) (see Additional file 5: Table S4). "*median difference compared to array*" was calculated as:

$$\frac{|median\, density\, of\, array\, background\, for\, tissue - median\, density\, of\, dmCpGs\, for\, tissue|}{median\, density\, of\, array\, background\, for\, tissue} \cdot 100$$

CGI status and genomic region analysis. CGI island membership was assigned to each probe using Illumina's 450K annotation with the R/Bioconductor package *IlluminaHumanMethylation450kanno.ilmn12.hg19* (version 0.6.0). Genomic region position was assigned using the R/Bioconductor packages *TxDb.Hsapiens.UCSC.hg19.knownGene* (version 3.2.2) and *ChIPseeker* (version 1.12.0)(Yu et al. 2015). Statistical significance with respect to concrete CGI status or genomic regions was determined by two-sided Fisher's tests (significance level $P<0.05$), and ORs were used as a measure of the association effect with respect to a particular feature (see Additional file 6: Table S5). Appropriate backgrounds which included all the probes interrogated by the HumanMethylation450 BeadChip array in each of the comparisons were used for statistical purposes.

DNA methylation age analyses. DNA methylation age of normal and tumoral samples was predicted by applying Horvath's age predictor as implemented in R/Bioconductor package *wateRmelon* (version 1.18.0)(Horvath 2013; Pidsley et al. 2013). The correlation of the predicted age thus obtained with chronological age was measured with the Pearson product-moment correlation coefficient using R package *stats* (version 3.3.3), which measures significance using a Student's-t sampling distribution.

Gene and KEGG ontology analyses. Gene and KEGG (Kyoto Encyclopedia of Genes and Genomes) pathway ontology enrichments were calculated using the R/Bioconductor package *missMethyl* (version 1.8.0, *gometh* function)(Phipson et al. 2016), which performs one-sided hypergeometric tests taking into account and correcting for any bias derived from differing numbers of probes per gene interrogated by the array (see Additional file 13: Table S12). The annotation databases that were interrogated are http://www.kegg.jp/kegg/rest/keggapi.html for KEGG ontology, and the R/Bioconductor package *GO.db* (version 3.4.1) for gene ontology purposes. Appropriate backgrounds of total probes for each given context were employed in the corresponding analyses.

SUPPLEMENTARY REFERENCES

Chen Y, Lemire M, Choufani S, Butcher DT, Grafodatskaya D, Zanke BW, Gallinger S,… Weksberg R (2013) Discovery of cross-reactive probes and polymorphic CpGs in the Illumina Infinium HumanMethylation450 microarray. *Epigenetics* 8, 203–209.

Du P, Kibbe WA & Lin SM (2008) lumi: a pipeline for processing Illumina microarray. *Bioinforma. Oxf. Engl.* 24, 1547–1548.

Horvath S (2013) DNA methylation age of human tissues and cell types. *Genome Biol.* 14, R115.

Houseman EA, Accomando WP, Koestler DC, Christensen BC, Marsit CJ, Nelson HH, … Kelsey KT (2012) DNA methylation arrays as surrogate measures of cell mixture distribution. *BMC Bioinformatics* 13, 86.

Leek JT & Storey JD (2007) Capturing Heterogeneity in Gene Expression Studies by Surrogate Variable Analysis. *PLOS Genet.* 3, e161.

Morris TJ, Butcher LM, Feber A, Teschendorff AE, Chakravarthy AR, Wojdacz TK & Beck S (2014) ChAMP: 450k Chip Analysis Methylation Pipeline. *Bioinforma. Oxf. Engl.* 30, 428–430.

Phipson B, Maksimovic J & Oshlack A (2016) missMethyl: an R package for analyzing data from Illumina's HumanMethylation450 platform. *Bioinformatics* 32, 286–288.

Pidsley R, Y Wong CC, Volta M, Lunnon K, Mill J & Schalkwyk LC (2013) A data-driven approach to preprocessing Illumina 450K methylation array data. *BMC Genomics* 14, 293.

Teschendorff AE, Marabita F, Lechner M, Bartlett T, Tegner J, Gomez-Cabrero D & Beck S (2013) A beta-mixture quantile normalization method for correcting probe design bias in Illumina Infinium 450 k DNA methylation data. *Bioinforma. Oxf. Engl.* 29, 189–196.

Touleimat N & Tost J (2012) Complete pipeline for Infinium(®) Human Methylation 450K BeadChip data processing using subset quantile normalization for accurate DNA methylation estimation. *Epigenomics* 4, 325–341.

Xu Z, Niu L, Li L & Taylor JA (2016) ENmix: a novel background correction method for Illumina HumanMethylation450 BeadChip. *Nucleic Acids Res.* 44, e20.

Yu G, Wang L-G & He Q-Y (2015) ChIPseeker: an R/Bioconductor package for ChIP peak annotation, comparison and visualization. *Bioinforma. Oxf. Engl.* 31, 2382–2383.

SUPPLEMENTARY TABLE LEGENDS

Table S1. Patient ID, sample information and relevant clinicopathological features obtained from TCGA consortium, Guintinalvo et al. (2013) and Bormann et al., (2016) datasets.

Table S2. Annotation and statistical significance of CpG probes differentially methylated in aging and cancer (dmCpGs). Effect size column reflects the mean- sva-corrected- M-value difference between compared groups. Methylstat column indicates the direction of the methylation change (hyper – hypermethylated in OLD compared to YOUNG individuals or hypermethylated in Primary tumors compared to Solid tissue normal; hypo – hypomethylated in OLD in comparison to YOUNG individuals or hypomethylated in Primary Tumor with respect to Solid Tissue Normal). It also includes dmCpGs obtained from additional analyses of lung and blood datasets, related to Additional file 1: Figure S5a.

Table S3. Statistical results obtained from Wilcoxon rank-sum non-parametric tests comparing M-value distributions of significant cancer versus aging dmCpGs, related to Fig. 1c.

Table S4. Statistical results obtained from Wilcoxon rank-sum non-parametric tests comparing CpG density distributions of significant dmCpGs in aging or cancer versus their corresponding background array distribution, related to Fig. 2a.

Table S5. Statistical results obtained from two-tailed Fisher's exact tests calculating CpG and gene location enrichments of significant dmCpGs in aging and cancer compared with their respective background array distribution, related to Figs. 2b and 2c.

Table S6. List of common cancer or age related dmCpGs across breast, kidney, thyroid, skin and glia tissues. Common cancer dmCpGs were obtained from the overlap between the five

tissues used in our study, while common age related dmCpGs represent the intersection of significant dmCpGs in at least three out of five tissues.

Table S7. Statistical results obtained from two-tailed Fisher's exact tests calculating probe-sets overlaps and Jaccard Indices, related to Fig. 3d.

Table S8. Histone mark enrichment analysis of dmCpGs in cancer and aging. Enrichments were calculated between the dmCpGs in each of the analyses and the full collection of Roadmap epigenomics hg19 regions integrated in LOLA extended software. Corresponding array backgrounds were used for the different comparisons. Related to Fig. 4. It also includes histone enrichments obtained from additional analyses of lung and blood datasets, related to Additional file 1: Figure S5b.

Table S9. Patient ID, sample information and relevant clinicopathological features of LUNG and BLOOD obtained from TCGA consortium and Hannum et al. (2013) datasets, related to Additional file 1: Figure S5a.

Table S10. Chromatin state enrichment analysis of dmCpGs in cancer and aging. Enrichments were calculated between the dmCpGs from the different analyses and the hg19 chromatin segmentation regions (ChromHMM, 18 states) obtained from Roadmap and ENCODE consortia. A custom LOLA database including information related to the chromatin states in the different tissues/cell lines and corresponding array backgrounds were used for the correct enrichment calculation. Related to Fig 5a and Additional file 1: Figure S7.

Table S11. Transcription factor binding site enrichment analysis of dmCpGs in cancer and aging. Enrichments were calculated between the dmCpGs in each of the comparisons and the collection of transcription factor binding datasets from ENCODE (hg19) integrated in LOLA core software. Related to Fig. 5b and Additional file 1: Figure S8.

Table S12. Gene ontology and KEGG enrichment analysis of dmCpGs in cancer and aging.

Enrichments were calculated between the dmCpGs obtained from the different datasets and the KEGG and GO databases (http://www.kegg.jp/kegg/rest/keggapi.html for KEGG ontology and the R/Bioconductor package GO.db. for GO ontology)

Table S13. Patient ID, sample information and relevant clinicopathological features of KIRC data collected from TCGA consortium, related to Fig 7. Table includes statistical results obtained from differential gene expression analyses performed for aging (age_groups 1 & 2 versus 5) or cancer (Primary Tumor versus Solid Tissue Normal) comparisons.

Table S14. List of pairwise correlations (Spearman correlation >0.9 or <-0.9) observed between either aging or cancer related dmCpGs and genes expressed in normal KIRC datasets obtained from the TCGA consortium. Correlation analyses were performed with those samples corresponding to normal conditions (Solid Tissue Normal) and which presented both methylation and gene expression data for a given case. Related to Fig. 7e.

SUPPLEMENTARY FIGURES

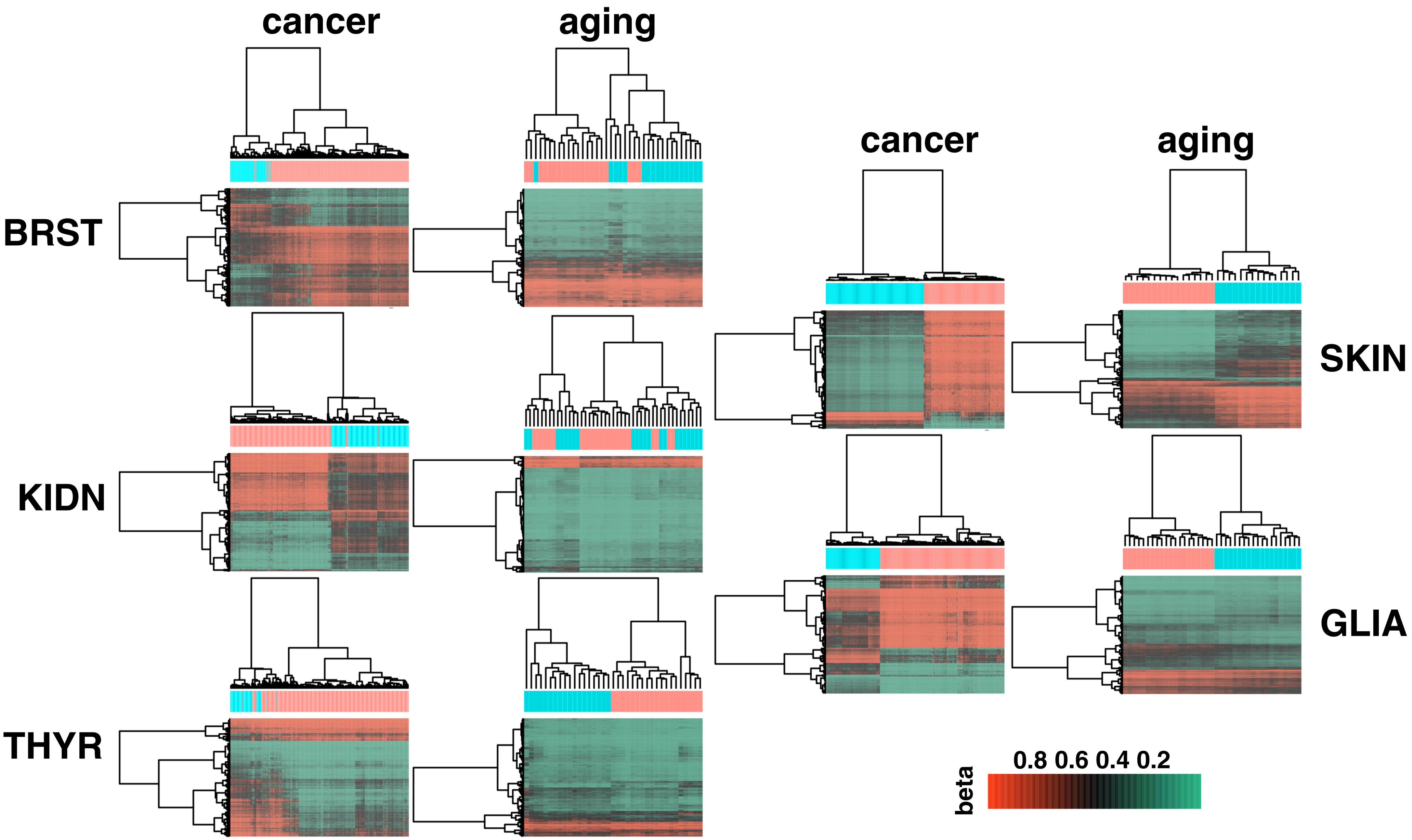

Figure S1. Hierarchical clustering and heatmaps including the 1,000 most significant dmCpGs for cancer and aging analyses. Methylation values are displayed from zero (green) to one (red).

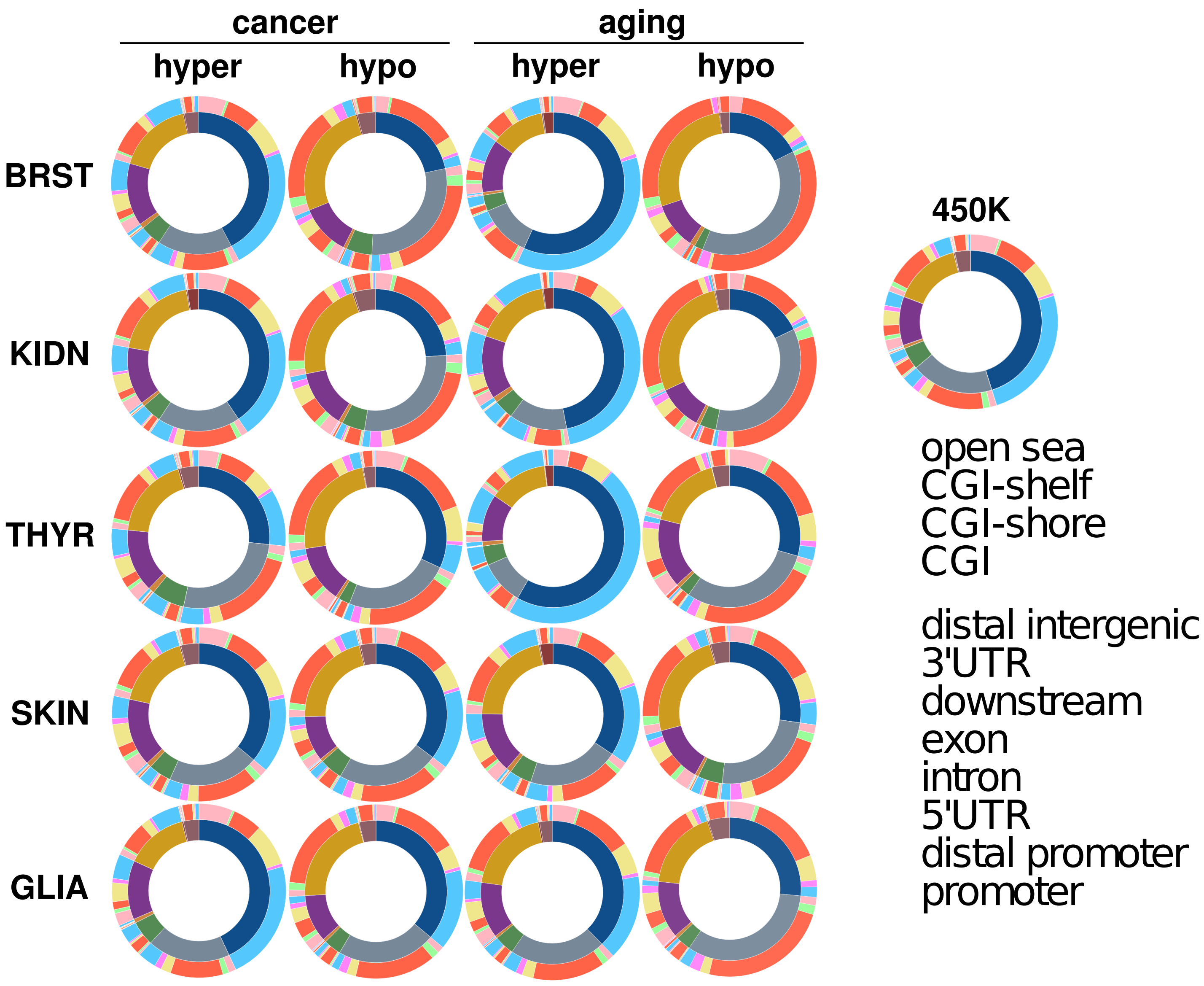

Figure S2. Sunburst plot charts representing integrated information of relative distribution of differentially methylated CpG according to their CpG island status (outer circle) and gene location status (inner circle).

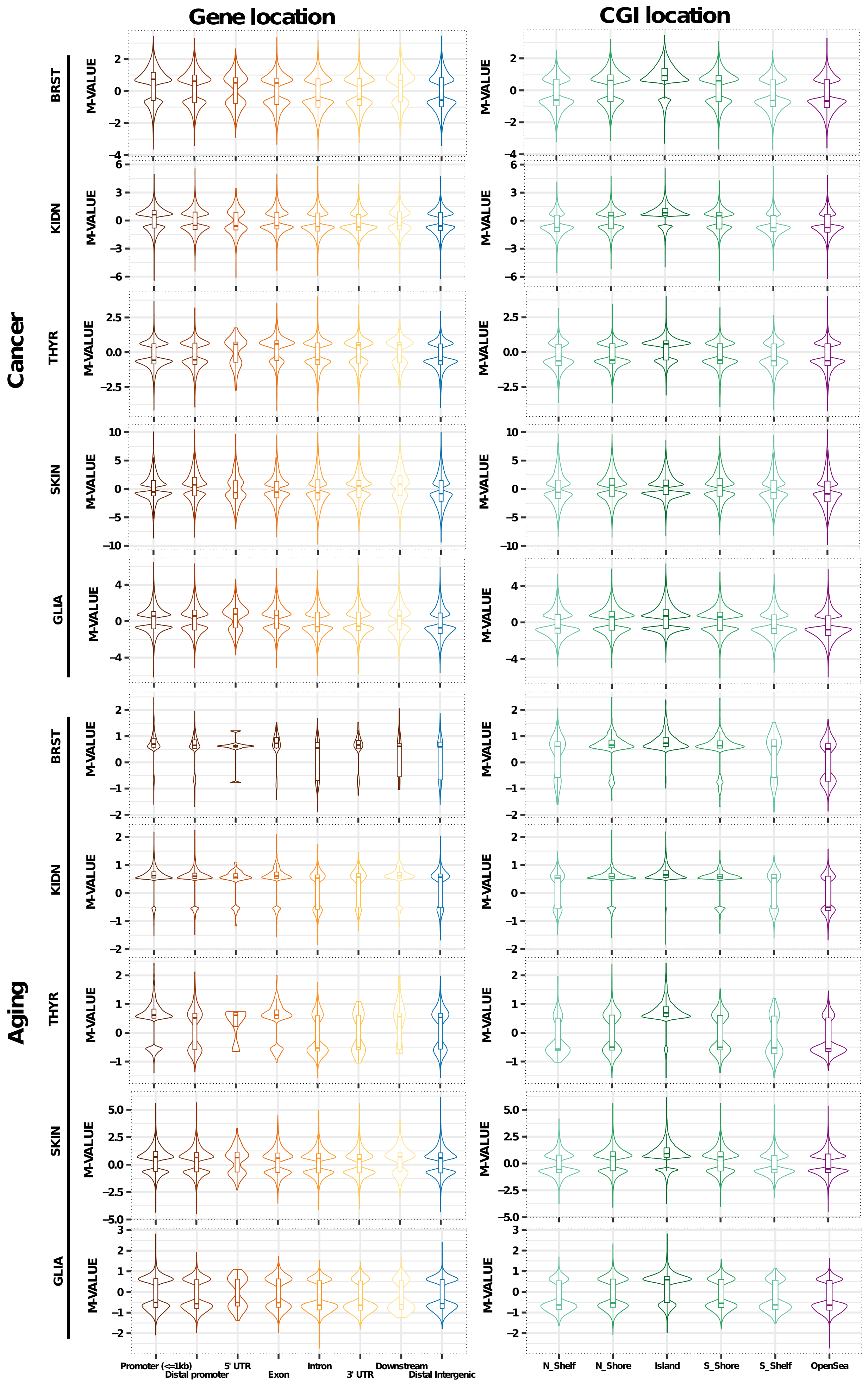

Figure S3. Violin plots representing magnitude of M-value methylation changes in cancer and aging, according to CpG island status (right plots) and gene location (left plots).

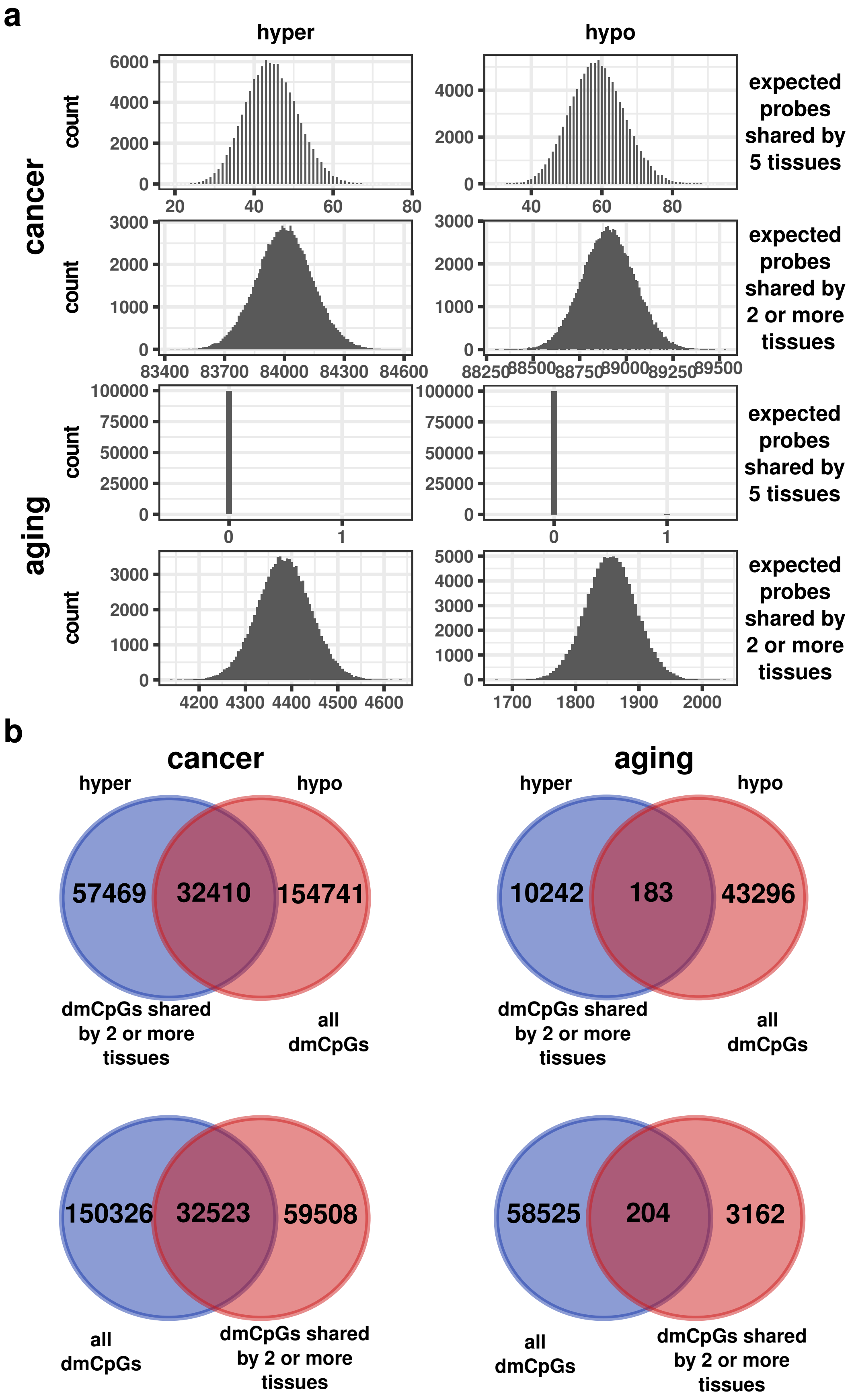

Figure S4. a Histograms showing obtained overlaps from 105 simulations of randomly extracting 5 sets of probes of sizes equal to the obtained dmCpGs of cancer and aging. b Overlap of hyper- and hypomethylated dmCpGs in aging and cancer. Venn diagrams showing the number and overlap total of non-redundant hyper- and hypomethylated dmCpGs detected in cancer and aging, when selecting dmCpGs shared by two or more tissues. Cancer Hyper Shared vs Hypo All: Fisher's test P<0.001, OR = 0.43, Expected hypergeometric mean, EHM = 46,506, Jaccard Index, JI = 0.13. Cancer HypoShared vs Hyper All: Fisher's Test P<0.001, OR = 0.43, EHM= 46,525, JI = 0.13. Aging Hyper Shared vs Hypo All: Fisher's Test P<0.001, OR = 0.13, EHM = 1,253, JI = 0.003. Aging HypoShared vs Hyper All: Fisher's Test P<0.001, OR = 0.33, EHM = 547, JI = 0.003.

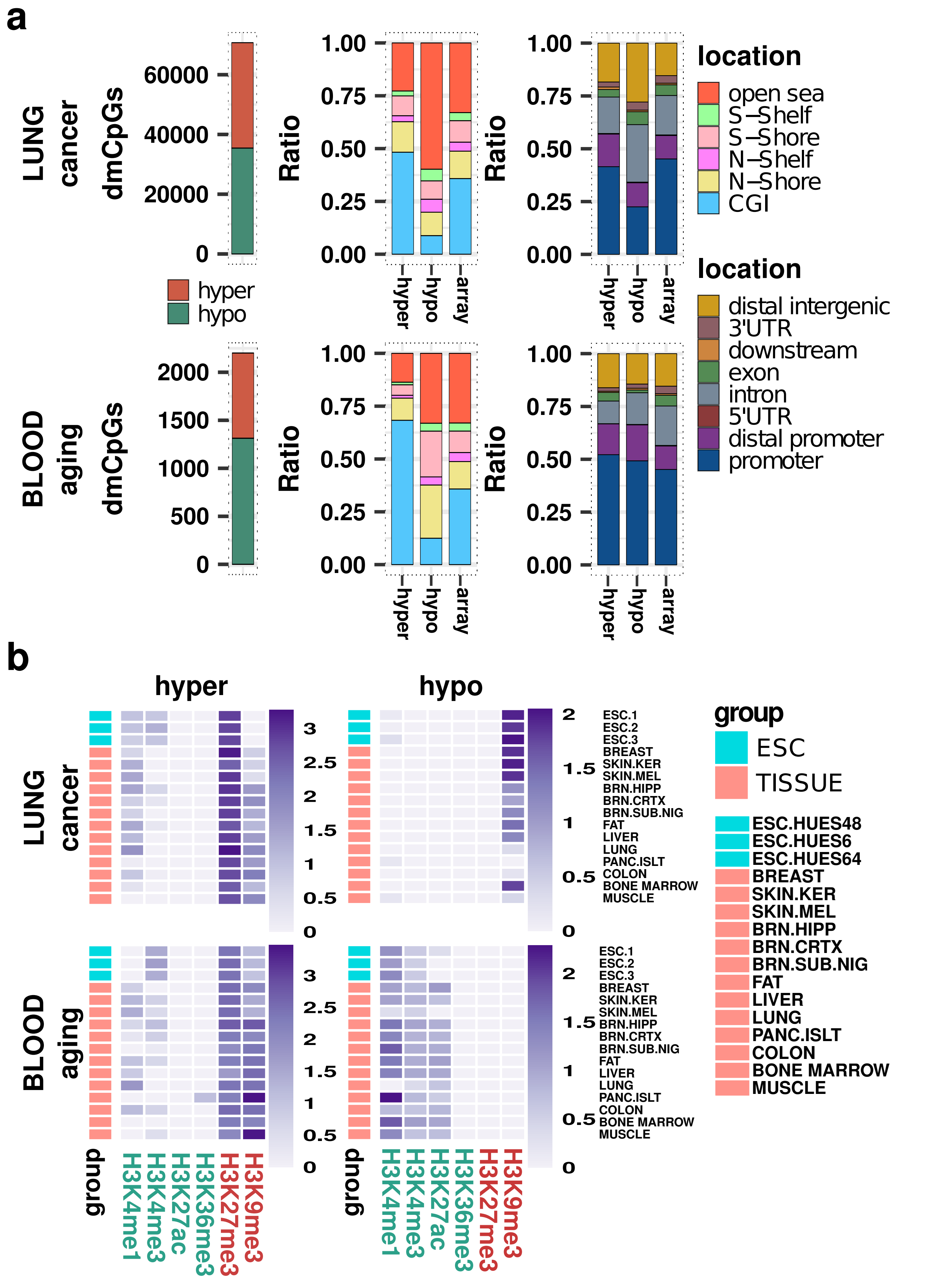

Figure S5. Additional lung and whole blood dataset analysis. a From left to right: stacked barplots indicating total number of dmCpGs detected in lung cancer and blood aging; stacked barplots indicating relative distribution of differentially methylated CpGs according to their CpG island status; stacked barplots indicating relative distribution of differentially methylated CpGs according to their gene location status. b Heatmaps depicting significant (P<0.05) over-enrichment of hyper- and hypomethylated dmCpG sites with different histone marks in aging and cancer, in a selection of 16 cell- and tissue-types (see Additional file 9: Table S8 for 98 full cell and tissue-types). Color code indicates the significant enrichment based on log2 odds ratio (OR).

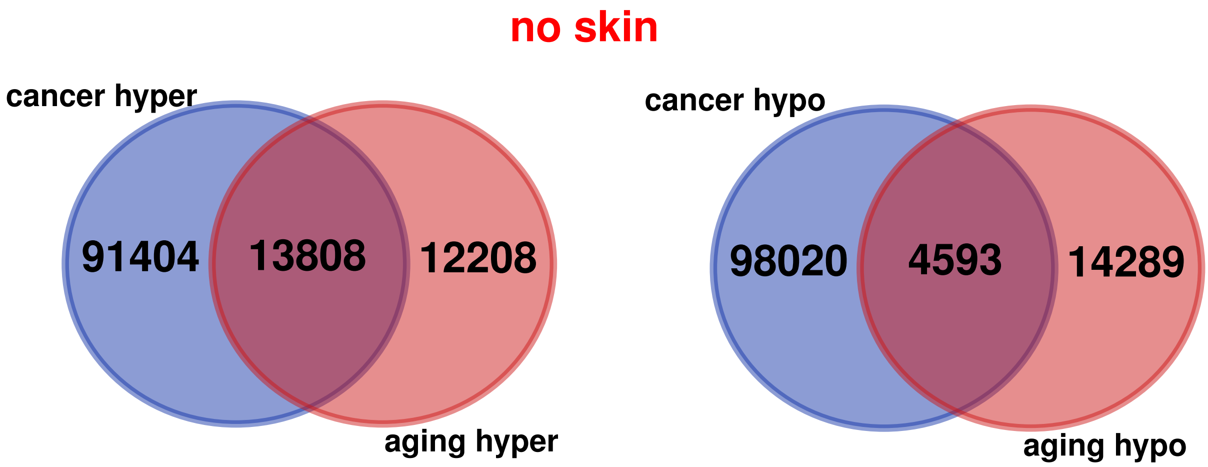

Figure S6. Venn diagrams showing the number and overlap of total non-redundant hyper- (left) and hypomethylated (right) dmCpGs detected in cancer and aging, without taking SKIN tissue into account. dmCpGs that were only hypermethylated or only hypomethylated between all tissues were chosen for the comparison. Fisher's tests, both P<0.001, ORs = 3.0 and 0.8, expected hypergeometric means = 7,568 and 5,357; JI = 0.12 and 0.05, respectively.

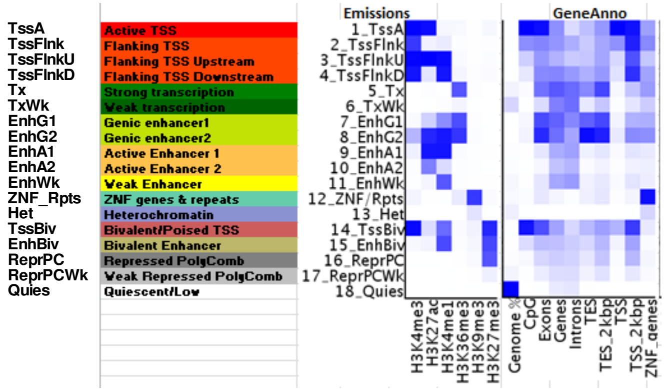

Figure S7. Heatmaps showing significant (P<0.05) over-enrichment of hyper- and hypomethylated dmCpG sites with different chromatin states in aging and cancer, in a selection of 16 cell- and tissue types (see Additional file 11: Table S10 for 98 full cell- and tissue-types). Color code indicates the significant enrichment based on log2 odds ratio (OR). On top of the figure, the NIH Roadmap learned ChromHMM model is displayed.

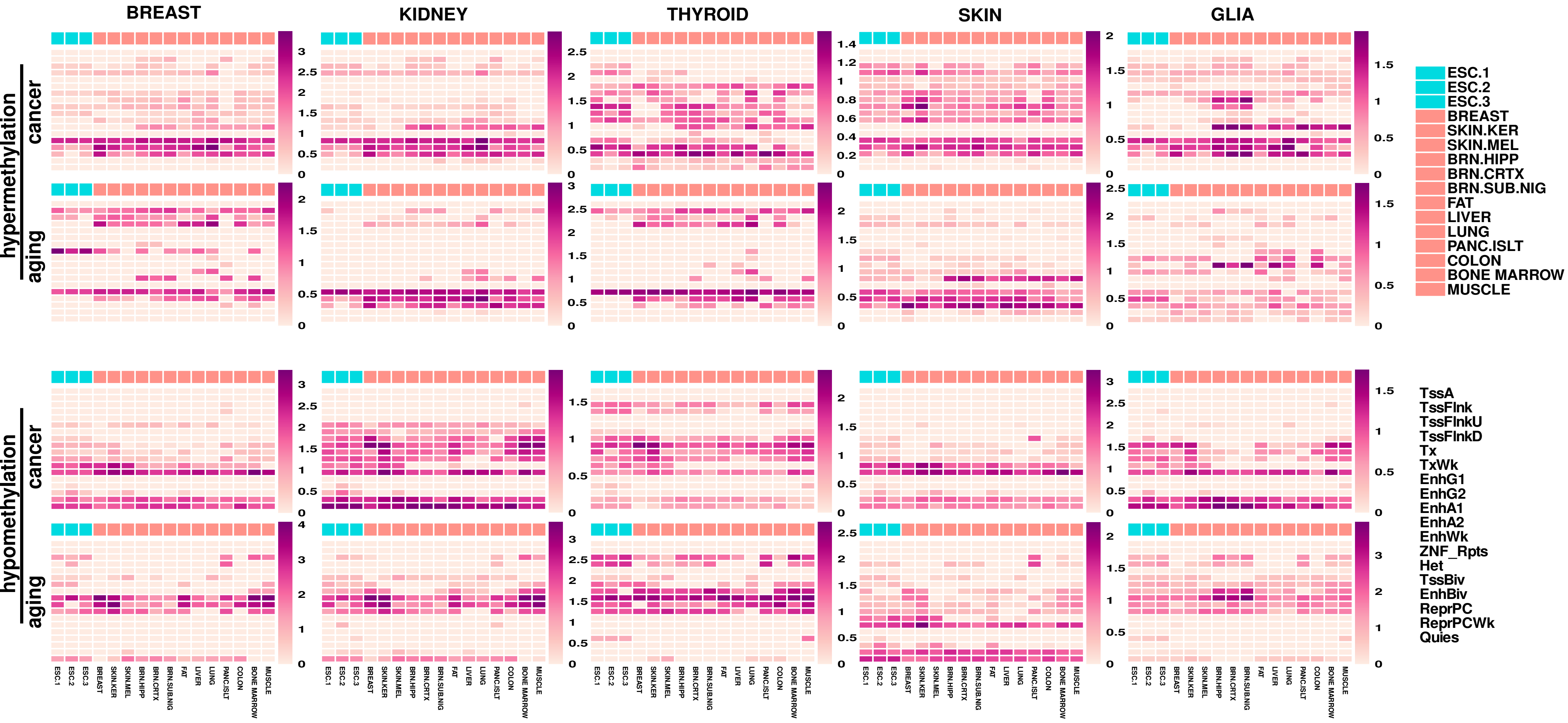

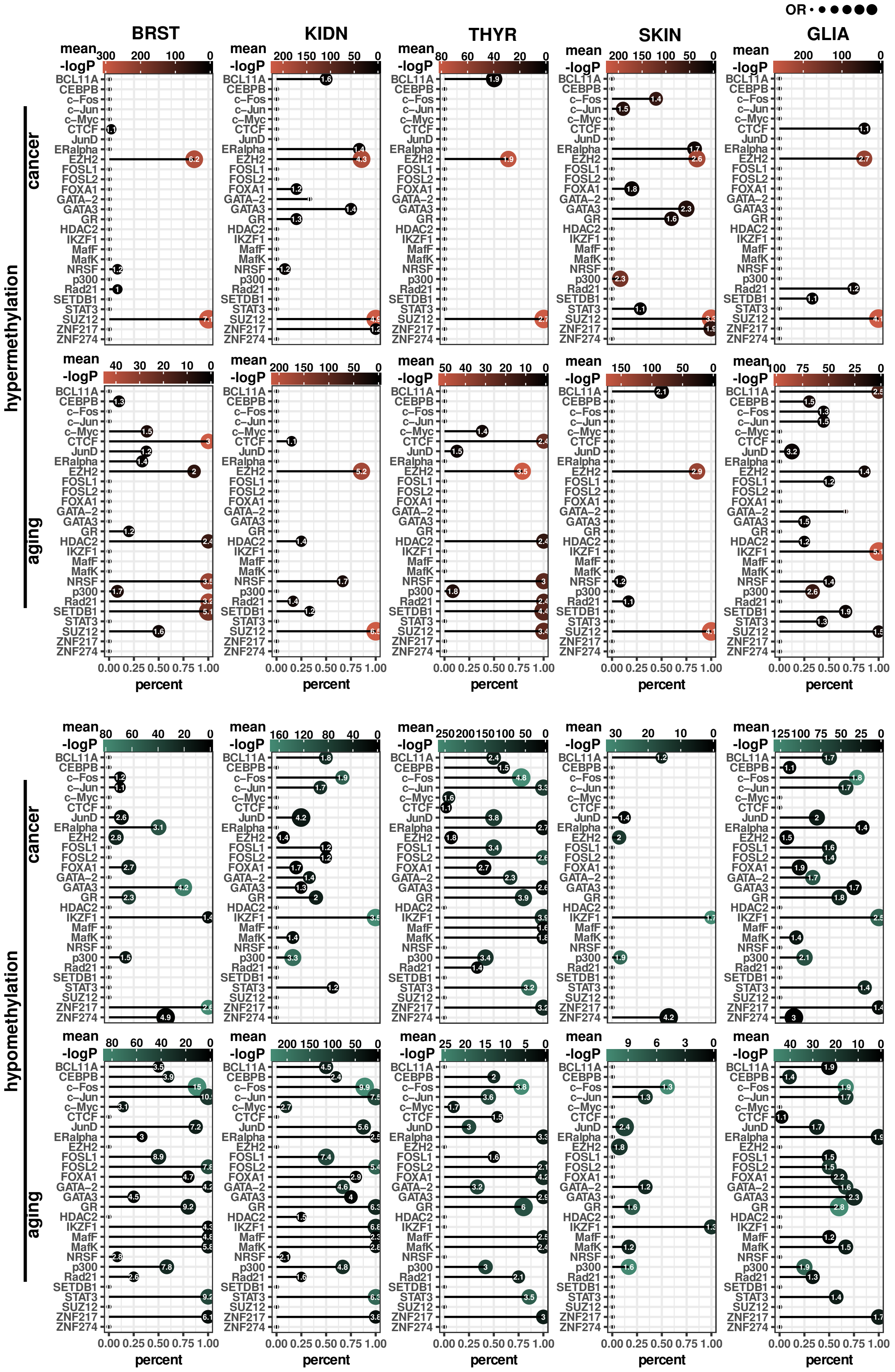

Figure S8. Lollipop plots showing the enrichment of different transcription factors at hyper- and hypomethylated dmCpG sites in aging and cancer. Color code indicates the significance of the over-enrichment based on log10 P-value. Sizes of circles and their corresponding numbers indicate enrichment based on odds ratio (OR). X-axis measures the percentage of possible tracks for which a significant enrichment was detected, as this is also a measure of enrichment. Only the most representative transcription factors (those that appeared as significantly over-enriched in at least 3 tracks or with an OR>3 in any track) were selected for data representation.

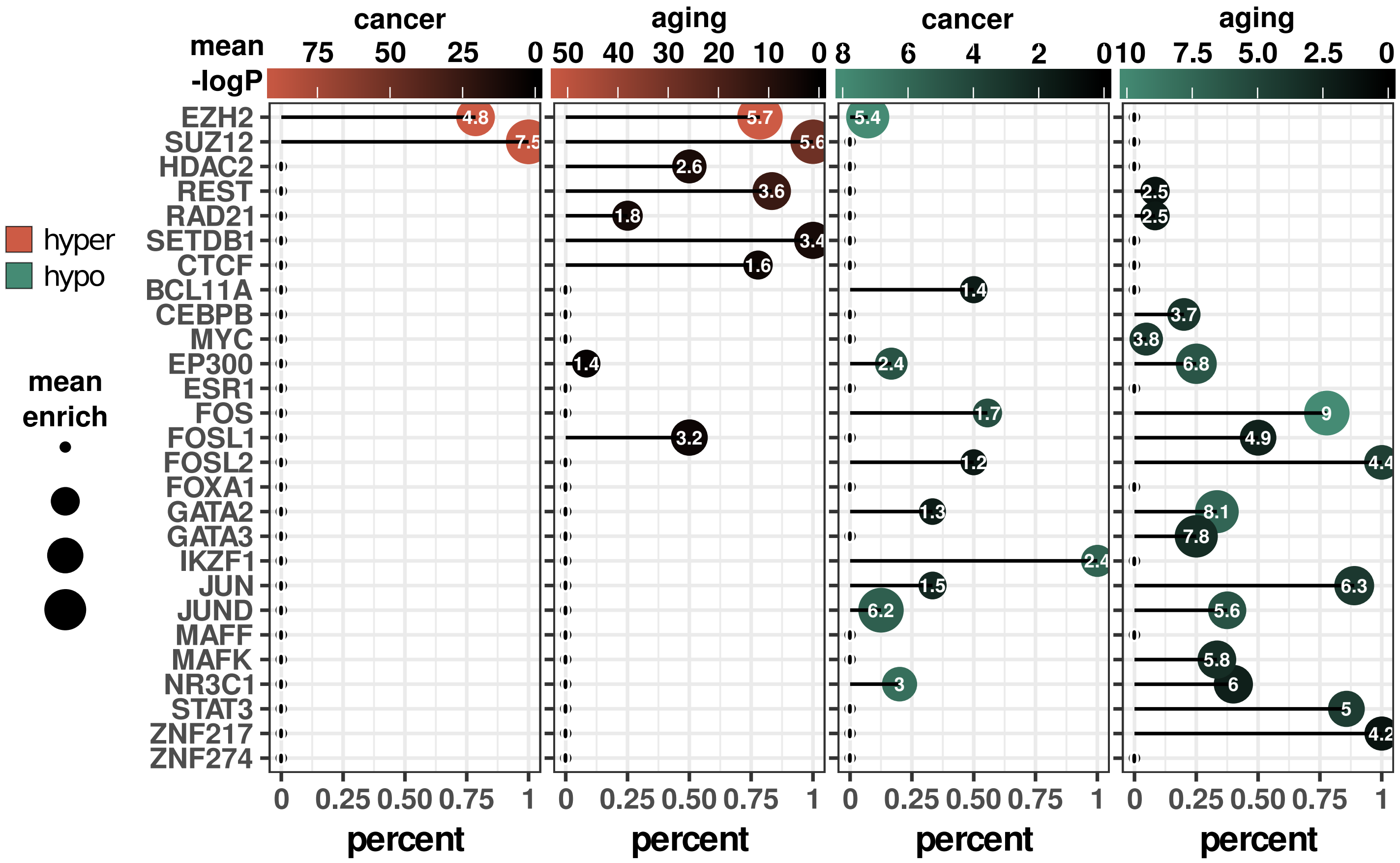

Figure S9. Lollipop plots showing the enrichment of different transcription factors for common hyper- and hypomethylated dmCpGs shared between 5 tissues for cancer (1,962 and 2,708 probes, respectively) or 3 tissues for aging (904 and 106 probes, respectively) (see Additional file 7: Table S6 for CpG lists). Color code indicates the significance of the over-enrichment based on log10 P-value. Sizes of circles and their corresponding number indicate enrichment based on odds ratio (OR). X-axis measures the percentage of possible tracks for which a significant enrichment was detected, as this is also a measure of enrichment.

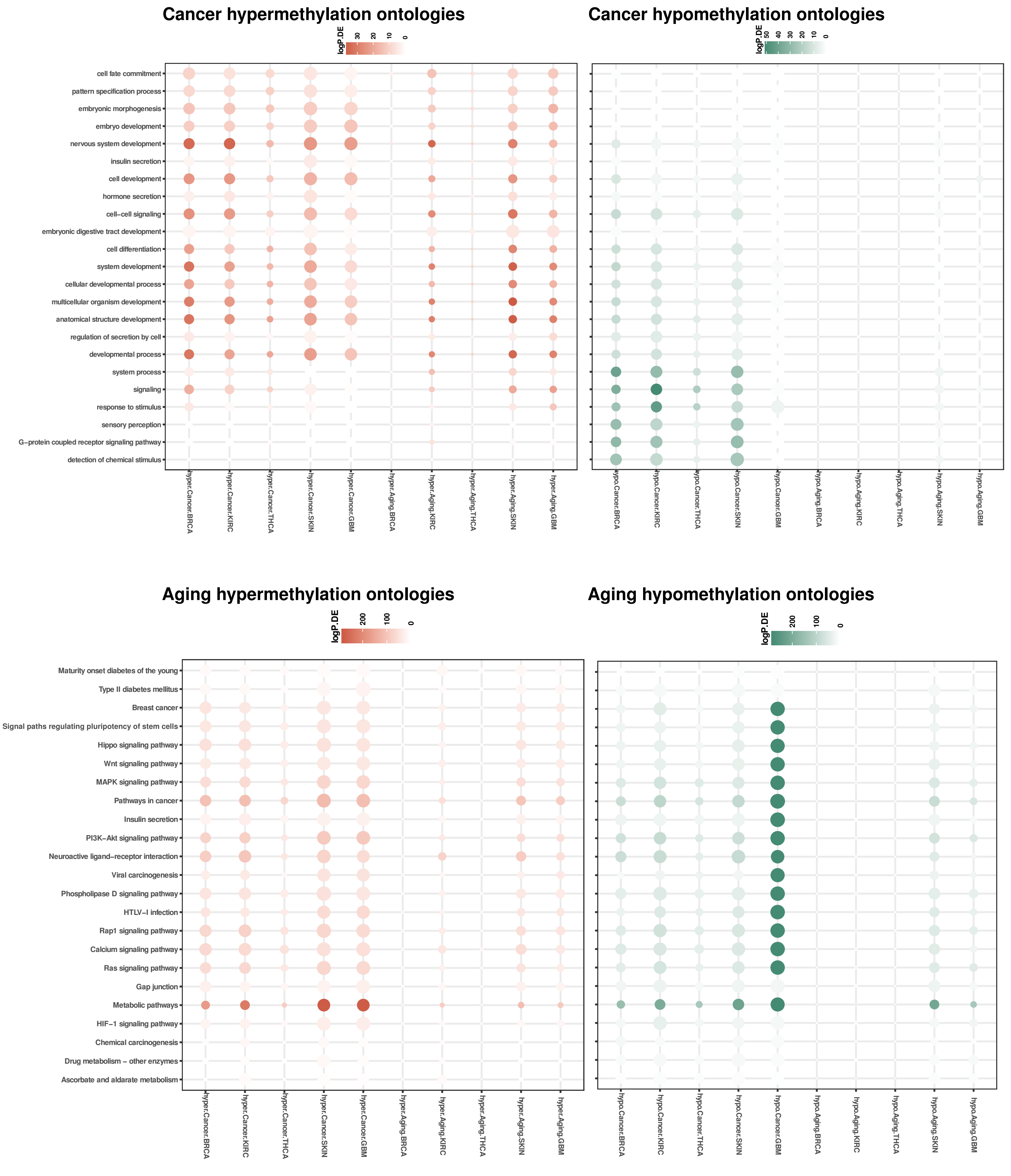

Figure S10. Panels indicating gene and KEGG pathway ontology enrichment for hyper- and hypomethylation dmCpGs in aging and cancer (see Additional file 13: Table S12 for detailed information).

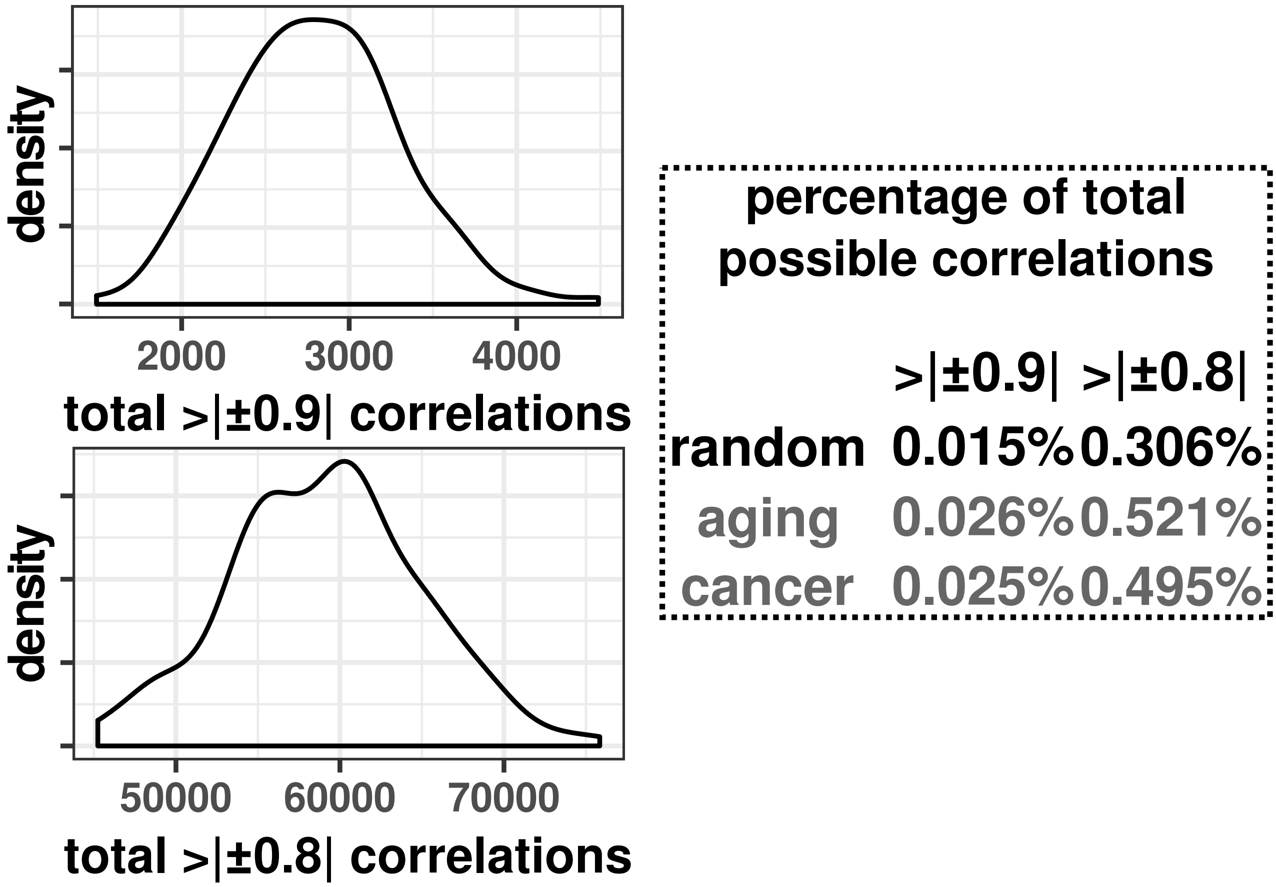

Figure S11. Density plots showing the number of Spearman correlations observed between DNA methylation and gene expression (> 0.8 or < -0.8) generated by randomly extracting 150 sets of 1000 probes from the KIRC 450K array and computing the correlations against all the genes expressed in the KIRC dataset across 18 normal kidney samples. On the right, the percentages of >|±0.8| correlations as compared to the total number of the computed correlations is shown.
